# Detection of the UV-vis silent biomarker trimethylamine-*N*-oxide via outer-sphere interactions in a lanthanide metal-organic framework

**DOI:** 10.1038/s42004-022-00690-8

**Published:** 2022-06-22

**Authors:** Hui Min, Zhonghang Chen, Zongsu Han, Kunyu Wang, Jun Xu, Wei Shi, Peng Cheng

**Affiliations:** 1 Department of Chemistry, Key Laboratory of Advanced Energy Materials Chemistry (MOE) and Renewable Energy Conversion and Storage Center (RECAST), College of Chemistry, Nankai University, Tianjin, 300071 China; 2 School of Materials Science and Engineering & National Institute for Advanced Materials Center for Rare Earth and Inorganic Functional Materials, Tianjin Key Lab for Rare Earth Materials and Applications, Nankai University, Tianjin, 300350 China

**Keywords:** Coordination chemistry, Materials for optics, Metal-organic frameworks, Sensors

## Abstract

Trimethylamine-*N*-oxide (TMAO) is a biomarker of the cardiovascular disease that is one of the leading causes of worldwide death. Facile detection of TMAO can significantly improve the survival rate of this disease by allowing early prevention. However, the UV-vis silent nature of TMAO makes it intricated to be detected by conventional sensing materials or analytical instruments. Here we show a bilanthanide metal-organic framework functionalized by borono group for the recognition of TMAO. Superior sensitivity, selectivity and anti-interference ability were achieved by the inverse emission intensity changes of the two lanthanide centers. The limit of detection is 15.6 μM, covering the clinical urinary concentration range of TMAO. A smartphone application was developed based on the change in *R*-*G*-*B* chromaticity. The sensing mechanism via a well-matched outer-sphere interaction governing the sensing function was studied in detail, providing fundamentals in molecular level for the design of advanced sensing materials for UV-Vis silent molecules.

## Abstract (web summary)

Detection of trimethylamine *N*-oxide (TMAO) can allow for early intervention of cardiovascular disease, but is challenging to achieve using conventional materials and instruments owing to it being spectroscopically silent in the UV-visible region. Here, a series of bilanthanide metalorganic frameworks functionalised with a borono group are shown to detect TMAO with high sensitivity and selectivity by exploiting the inverse emission intensity changes of the two lanthanide centres.

## Introduction

Convenient recognition of biomarkers for early diagnosis of major diseases of human beings is a general strategy that requires continuous development^1^. Cardiovascular disease (CVD) is one of the leading causes of death around the world and hence is a major public health problem^2^. Early detection of CVD is extremely important for reducing mortality because of the suddenness of this disease^3^. Trimethylamine-*N*-oxide (TMAO), an intestinal gut flora metabolite of phosphatidylcholine and *L*-carnitine, has been recognized as an important biomarker for CVD^4–6^. TMAO is mostly eliminated through urine, and the concentration of TMAO in infarcted patients is approximately 2.2 times higher than that in healthy people^7–9^. Therefore, real-time monitoring of TMAO concentrations in urine is highly important for CVD prevention. To date, there have been limited reports on TMAO recognition, and most of them have to use time-consuming and cumbersome chromatography or mass spectrometry^10,11^. Although electrochemical methods^12^, indicator displacement assays^13^ and colorimetric sensor arrays^14^ have been reported for sensing UVsilent biomarkers such as TMAO (Supplementary Table 1), there are deficiencies in the accuracy and convenience of such methods that have to be improved to satisfy the practical requirements. The development of a facile sensing material for quantitative recognition of TMAO to meet the requirement for early clinical diagnosis of CVD is highly desirable^3^. However, TMAO has no characteristic absorption or emission in the range of 200–800 nm (Supplementary Fig. 1), making it difficult to be detected via well-known sensing mechanisms^15,16^.

Owing to the designable structures, metal-organic frameworks (MOFs) have received great attention as sensing materials, which are able to match the targeted chemical analytes in both structures and energy levels^17–21^. Well-designed lanthanide MOFs have shown excellent potential in this field because of their outstanding luminescent properties from lanthanide centers with long lifetimes and sharp line emissions^22–25^. The sensing functions of high sensitivity, high selectivity and low systematic error can be achieved by lanthanide MOFs with two emission centers because of their excellent self-calibration function and color gradient feature originated from the multi-emission centers^26–31^. To achieve high-performance recognition for UV-vissilent TMAO, a well-matched host-guest interaction between the inner and outer coordination sphere in lanthanide MOFs is essential^32^. Since TMAO is an electron donor, the functionalization of lanthanide MOFs by an electron acceptor is a rational approach, but it has not been realized in MOF chemistry.

In this contribution, a family of lanthanide MOFs, {[Ln_2_(BIPA)_3_(EG)(H_2_O)_2_]·1.5DMA·6H_2_O}_n_ (Ln = Tb_x_Eu_1-x_, x = 1, 0.87, 0.80, 0.76, 0.67, 0.44, 0.42, 0.35, 0.08 and 0 for **B1**-**B10**, respectively; Ln = Gd for **B11**; H_2_BIPA = 5-boronoisophthalic acid, EG = ethylene glycol, DMA = *N*,*N-*dimethylacetamide) were synthesized for TMAO recognition. Borono-functionalized H_2_BIPA was used as both the linker and functional unit to interact directly with TMAO for the construction of lanthanide MOFs because: i) H_2_BIPA has suitable molecular energy levels that can sensitize both Eu^3+^ and Tb^3+^ ions for fluorescence through the antenna effect; ii) the borono group is electron-deficient and hence can interact with the terminal oxygen of TMAO (Supplementary Fig. 2), influencing the energy transfer process and hence changing the emission intensity and color. However, no lanthanide MOFs with H_2_BIPA has been reported thus far^33–37^. **B1**-**B10** were synthesized to optimize the sensing function for TMAO, while **B11** and {[Tb_0.92_Eu_1.08_(IPA)_3_(EG)_2_]·H_2_O}_n_ (**B12**, H_2_IPA = isophthalic acid) without a borono group were synthesized to study the recognition mechanism. By a well-designed mixed lanthanide strategy, highly selective and sensitive detection of TMAO in simulative urine was achieved by **B7**, based on which a facile smartphone application was successfully developed.

## Results and Discussion

### Structure and characterizations

**Fig. 1:**
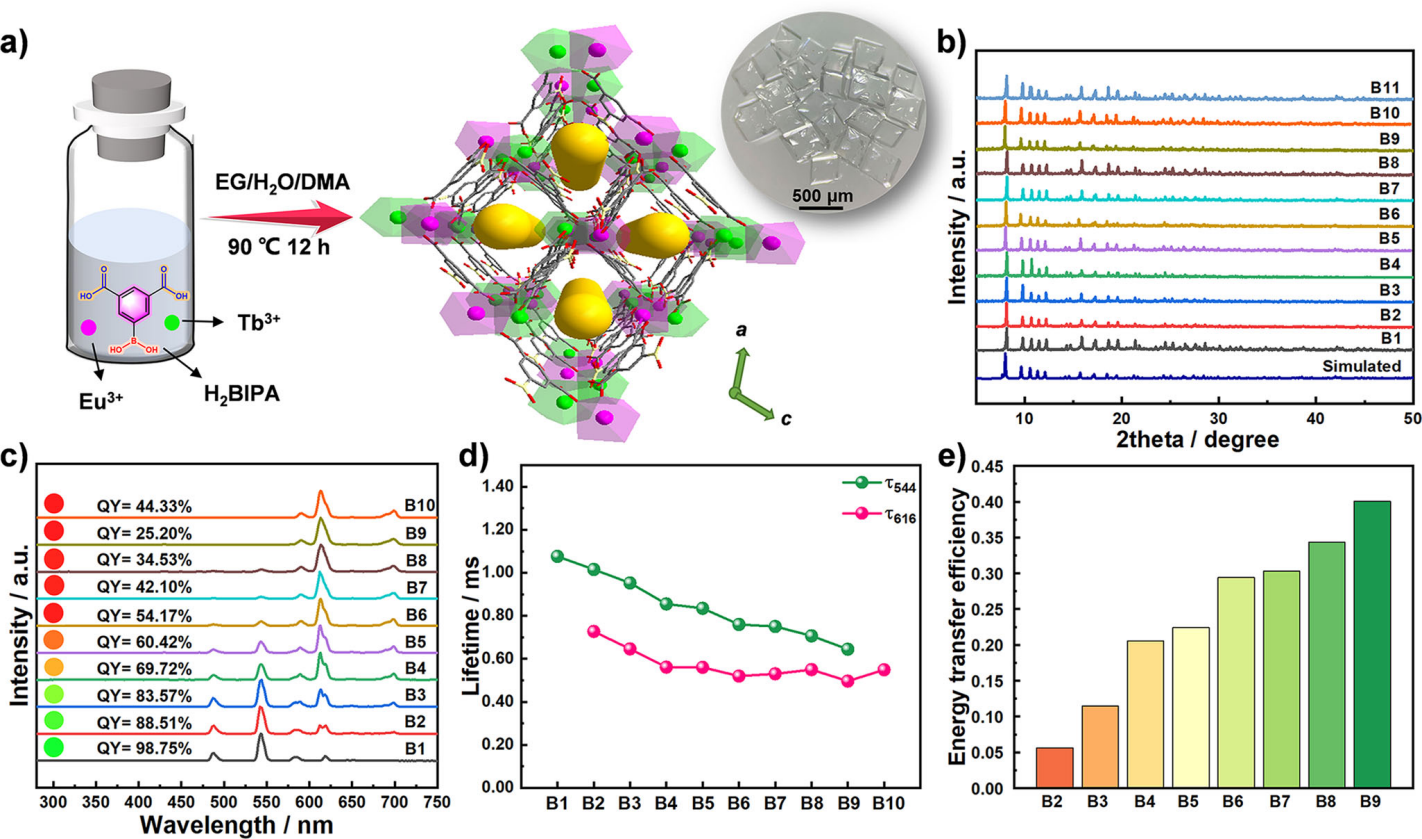
Synthesis and luminescent properties of B1-B11. **a** Synthesis and structure of **B7** with its photo under natural light. Atom codes: Tb (green), Eu (pink), C (gray), B (yellow) and O (red). Partial hydrogen atoms and solvent molecules are omitted for clarity. **b** PXRD of **B1**-**B11**. **c** Solid-state fluorescence spectra of **B1**-**B10** excited at 254 nm. The pictures of **B1**-**B10** were obtained under 254 nm UV light. QY is absolute quantum yield. **d** Solid-state lifetimes of **B1**-**B10** excited at 254 nm. **e** Energy transfer efficiencies of **B2**-**B9**.

Single-crystal *X*-ray diffraction studies showed that isostructural **B1**-**B11** crystallized in the *P*2_1_/*c* space group (Supplementary data 1–3 and Supplementary Table 2). The structure of **B1** is described here as a representative. The asymmetric unit consists of two crystallographically independent Tb^3+^ ions, three BIPA^2−^ ions, two water molecules and one EG molecule in the coordination sphere and one and a half DMA and six water molecules in the lattice (Supplementary Fig. 3a). Both Tb^3+^ ions are eight-coordinated but have different coordination environments: bicapped trigonal prism and triangular dodecahedron (Supplementary Fig. 3b). Tb1 is coordinated by six oxygen atoms from five BIPA^2−^ moieties and two oxygen atoms from two water molecules; Tb2 is coordinated by six oxygen atoms from five BIPA^2−^ moieties and two oxygen atoms from an EG molecule. The Tb-O bond lengths range from 2.309(3) to 2.832(4) Å. Tb1 and Tb2 are bridged by one *μ*_2_-*η*^2^:*η*^1^-carboxylate and three *μ*_2_-*η*^1^:*η*^1^-carboxylates from four BIPA^2−^ moieties to form a binuclear unit with a Tb···Tb distance of 4.045(4) Å (Supplementary Fig. 3c). The binuclear units are connected by BIPA^2−^ to form two-dimensional networks in the *ab* plane (Supplementary Fig. 3d), which are further connected by BIPA^2−^ along the *c* axis to form a three-dimensional framework (Fig. 1a). Hydrogen bonds are formed by the lattice water molecules and the coordinated EG molecules, water molecules and borono groups (Supplementary Fig. 3e). By viewing BIPA^2−^ as 2-connected linker and the binuclear unit as 6-connected nodes, **B1** can be simplified as a new 2,6-*c* net with a Schläfli symbol of {8^12^;12^3^}{8}_3_ (Supplementary Fig. 3f).

The morphology observed from scanning electron microscope (SEM) images and Fourier transform infrared (FTIR) spectra of **B1**-**B11** are almost the same (Supplementary Fig. 4), and the powder *X*-ray diffraction (PXRD) patterns of **B1**-**B11** are well consistent with the simulated peaks based on the single-crystal data of **B1** (Fig. 1b), confirming the high-phase purity of these compounds. The water stability at different pH values of representative **B1** was studied by FTIR and PXRD analysis (Supplementary Fig. 5), indicating that **B1** is stable in the pH range of 3–12. This high stability is attributed to hydrogen bond networks of lattice water molecules in the rigid framework, which increases the steric hindrance and prevents water molecules from attacking the Tb-O bond^38,39^. The thermogravimetric analysis (TGA) of **B1**-**B11** showed similar weight losses in the temperature range of 40–800 °C (Supplementary Fig. 6). Taking **B1** as an example, the first weight loss is from the release of six water molecules (calc. 8.44%, obs. 8.65%) in the range of 40–120 °C. A weight loss of 9.50% was observed from 120 to 320 °C, corresponding to the loss of one DMA and two coordinated water molecules (calc. 9.62%). The third weight loss of 8.08% was observed between 320 and 450 °C, indicating the absence of half a DMA molecule and one coordinated EG molecule (calc. 8.25%). Further weight losses were attributed to the decomposition of the framework.

### Luminescent properties

The solid-state luminescence spectra of **B1**-**B10** were acquired at room temperature (Fig. 1c). Four well-resolved emission peaks at 488, 544, 583 and 621 nm were observed in the spectrum of **B1** that can be assigned to the ^5^D_4_ → ^7^F_J_ (J = 6, 5, 4, 3) transitions of the Tb^3+^ ion. The characteristic emissions at 593, 616, 653 and 703 nm in the spectrum of **B10** are attributed to the ^5^D_0_ → ^7^F_J_ (J = 1, 2, 3, 4) transitions of Eu^3+^ ions. The spectra of **B2**-**B9** simultaneously show emission peaks attributed to Eu^3+^ and Tb^3+^, indicating successful mixing of the two lanthanide ions. The lifetimes of excited states ^5^D_4_ (Tb^3+^) and/or ^5^D_0_ (Eu^3+^) in **B1**-**B10** were measured to study the Tb^3+^-to-Eu^3+^ energy transfer process (Supplementary Figs. 7, 8). Compared with the lifetime of **B1** (^5^D_4_), the lifetime of Tb^3+^ in **B2**-**B9** is shorter (Fig. 1d), which might be caused by the formation of a new energy transfer pathway (Supplementary Figs. 9, 10). The efficiency of energy transfer (*E*) can be quantitatively described using *E* = 1 - τ_Tb-Eu_/τ_Tb_^40^, where τ_Tb-Eu_ and τ_Tb_ are the excited-state lifetimes of Tb^3+^ in **B2**-**B9** and **B1**, respectively (Fig. 1e). The calculated *E* values reached 40% when the Eu content reached 90% (Supplementary Table 3), further proving the occurrence of the Tb^3+^-to-Eu^3+^ energy transfer process in **B2**-**B9**. The quantum yields of **B1** and **B10** were determined to be 98.75 and 44.33%, respectively (Supplementary Fig. 11). With increasing Eu content, the quantum yields of **B2**-**B9** gradually decreased because of the additional energy transfer pathway from Tb^3+^ to Eu^3+^.

### Detection of TMAO

**Fig. 2:**
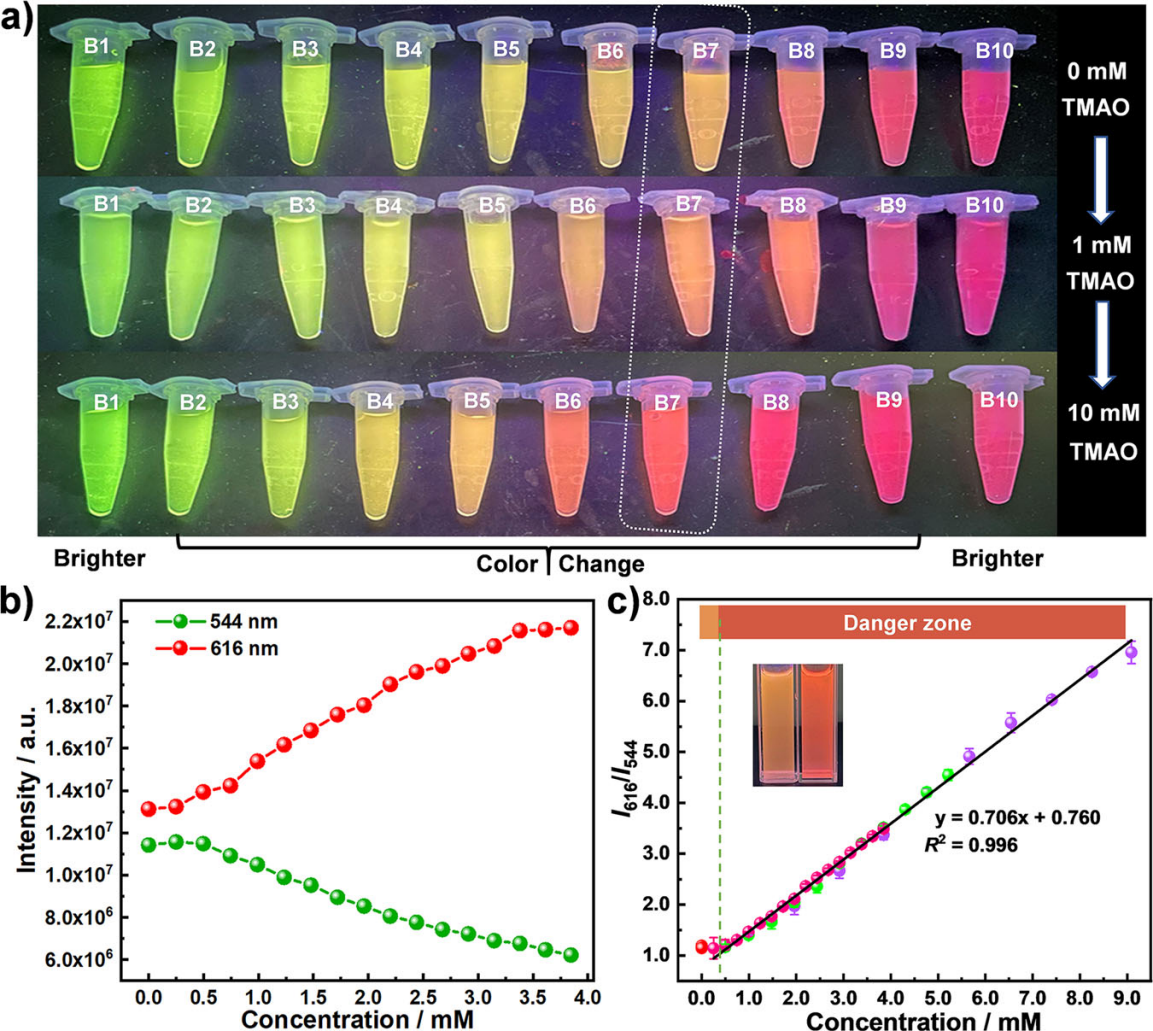
Detection of TMAO. **a** Luminescent responses of **B1**-**B10** aqueous dispersions toward 1 mM and 10 mM TMAO under a 254 nm UV lamp. **b** Luminescent intensities of **B7** at 544 and 616 nm with additions of TMAO. **c** Integrated luminescent intensities of **B7** toward TMAO with different concentrations; green dotted line shows the critical TMAO concentration in infarcted patients and the inset shows photographs of **B7** before and after adding 9 mM TMAO under 254 nm UV light. Error bar shows the data standard deviation of measured and theoretical values.

A screen of **B1**-**B10** for the detection of TMAO was performed. When 1 mM or 10 mM TMAO was added, different degrees of color changes of the aqueous dispersions of **B1–B10** were observed (Fig. 2a). Fluorescence titration experiments of TMAO were first performed for **B1** and **B10** with a single lanthanide center. The fluorescence intensities of **B1** and **B10** increased after the addition of TMAO (Supplementary Fig. 12). The emission intensities in the spectrum of **B1** at 544 nm and the spectrum of **B10** at 616 nm follow the Benesi–Hildebrand equation: *I*_0_/(*I*-*I*_0_) = *K*_BH_/[*C*] + b^41,42^, where *I*_0_ and *I* are the emission intensities without or with TMAO, *K*_BH_ is the association constant, and [*C*] is the concentration of TMAO. The *K*_BH_ values were calculated to be 1.57×10^4 ^M and 1.75×10^4 ^M for **B1** and **B10**, respectively. These large *K*_BH_ values indicate an effective interaction between **B1**/**B10** and TMAO. Due to the isostructural nature of these compounds, the same interaction should exist in **B2**-**B9** and influence the energy transfer process to induce the color change of the aqueous dispersions of **B2**-**B9**. The ratio-metric fluorescence responses of **B2-B9** toward different concentrations of TMAO were studied (Supplementary Fig. 13). **B9** was found to be most sensitive to TMAO. However, the deep red color of **B9** is not conducive to the visual detection of TMAO. Considering that **B4**, **B5**, **B6**, and **B7** can all be applied for visual detection of TMAO and that **B7** shows the best comprehensive performance, the use of **B7** for the detection of TMAO was further studied in detail.

The water stability of **B7** at different pH values was studied by FTIR and PXRD (Supplementary Fig. 14), indicating that **B7** is stable in the pH range of 3–12. Considering that the specific lanthanide distribution can affect the energy transfer efficiency between Tb^3+^ and Eu^3+^, thus affecting the detection result^27,28,43^, elemental mapping of **B7** has been investigated (Supplementary Fig. 15). Elements of B, Eu and Tb are uniformly distributed in **B7**, indicating the arrangement of Tb and Eu atoms in **B7** are controllable.

Aqueous dispersions containing different concentrations of **B7** (0.15–0.30 mg/mL) showed stable emission intensity ratios of *I*_616_/*I*_544_ (Supplementary Fig. 16), indicating that water does not interfere with the fluorescence sensing results. The lack of obvious changes in the peak intensities in the time-dependent luminescence spectra of **B7** (Supplementary Fig. 17) excludes the influence of particle aggregation or structure change, indicating the high stability of **B7** in water. Luminescent titrations of TMAO to **B7** aqueous dispersions were performed (Fig. 2b). With the addition of TMAO, the fluorescence intensities at 544 nm decrease, while those at 616 nm increase. The color of the aqueous dispersions of **B7** changes from light yellow to pink after the addition of 9 mM TMAO under a 254 nm UV lamp (Fig. 2c, inset). The *I*_616_/*I*_544_ ratios exhibit a good linear relationship with the concentration of TMAO. To improve the detection accuracy, three independent fluorescence titrations with different volumes of TMAO solutions were performed (Supplementary Fig. 18). A combined result of these three titrations is shown in Fig. 2c, obeying the equation *I*_616_/*I*_544_ = 0.706[*C*] + 0.760. The limit of detection was calculated to be 15.6 μM, which is lower than the clinical urinary TMAO concentration^9^.

**Fig. 3:**
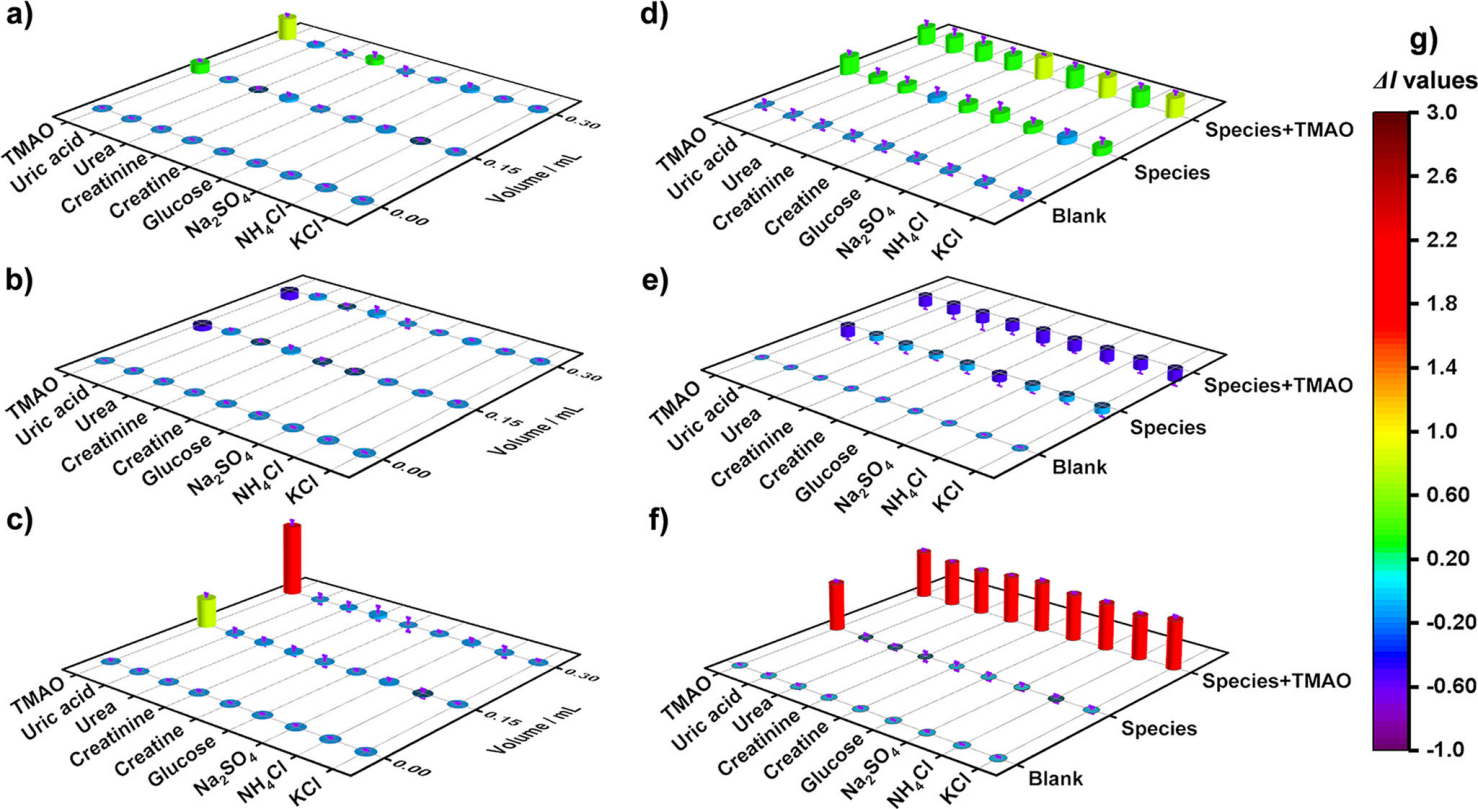
Selectivity and anti-interference ability of B7. Selectivity of **B7** aqueous dispersions towards TMAO (3.25 mM) in common urine disruptors based on the change of luminescent intensities at 544 nm **a**, 616 nm **b**, and the ratio of *I*_616_/*I*_544_ **c**, respectively. Anti-interference ability of **B7** aqueous dispersions in common urine disruptors without/with 3.25 mM TMAO based on the change of luminescent intensities at 544 nm **d** or 616 nm **e**, and the ratio of *I*_616_/*I*_544_ **f**, respectively. **g** Indication by the colormap. The concertation of creatine, creatinine glucose, uric acid, urea, KCl, NH_4_Cl and Na_2_SO_4_ was 1.33, 1.33, 1.33, 0.16, 1.33, 2.67, 2.67 and 1.33 mM, respectively. Error bar shows the standard deviation of three consecutive test values.

Selectivity and anti-interference experiments toward common urine disruptors, including creatine, creatinine, glucose, uric acid, urea, KCl, NH_4_Cl and Na_2_SO_4_, were performed (Fig. 3). After adding these interferents, the fluorescence intensities at 544 nm or 616 nm exhibited moderate selectivity (Fig. 3a, b) but poor anti-interference ability (Fig. 3d, e). However, when using the ratio of *I*_616_/*I*_544_ as indicator, excellent selectivity and anti-interference ability can be simultaneously obtained (Fig. 3c, f), reflecting the superior advantage of the dual-emission center approach.

Due to the obvious discoloration to allow detection of TMAO, a smartphone application based on *R*-*G*-*B* chromaticity was proposed (Supplementary Fig. 19). Upon increasing the concentration of TMAO, real-time recording of the *R*-*G*-*B* chromaticity of **B7** was performed by a smartphone to determine the concentration-dependent *R*, *G* and *B* values (Supplementary Table 4). A linear relationship in the range of 0–10.7 mM was obtained based on the *R*/(*G* + *B*) value and the concentration of TMAO ([*C*]) according to the equation *R*/(*G* + *B*) = 0.0500[*C*] + 0.638. This application provides a simple on-site visible detection method for large-scale TMAO detection by taking advantage of the ubiquity of smartphones.

### Mechanism study

To clarify the sensing mechanism via the designed outer-sphere interaction, a series of characterizations and analyses were performed. PXRD of **B7** immersed in 10 mM TMAO for 12 h was consistent with that of the original sample (Supplementary Fig. 20), indicating the stability of **B7** in the presence of TMAO. The UV-vis absorption spectrum of TMAO does not overlap with the excitation and emission spectra of **B7** (Supplementary Fig. 21), excluding the possibility of an internal filtration effect and fluorescence resonance energy transfer mechanism^15,16^. According to the DFT calculation at the B3LYP/6-31 G* level (Supplementary Fig. 22), the LUMO energy level of TMAO is higher than that of H_2_BIPA, indicating the absence of photoinduced electron transfer progress^44^.

**Fig. 4:**
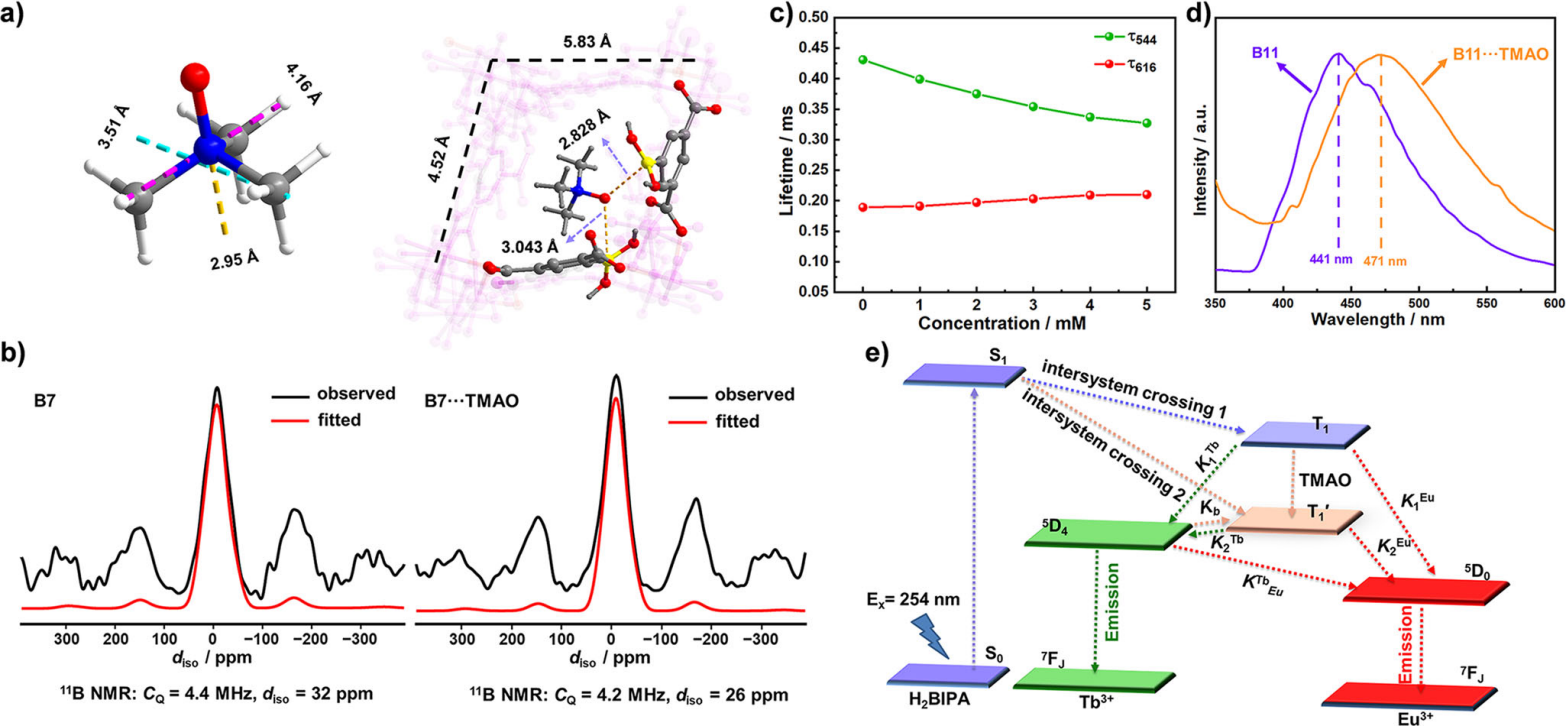
Direct interaction between B7 and TMAO. **a** Molecular size of TMAO and the interaction between TMAO and **B7**. **b** Solid-state MAS ^11^B NMR spectra of **B7** before and after treated by 100 mM TMAO for 24 h. **c** Fluorescent lifetimes at 616 nm (red) and 544 nm (green) of **B7** suspension at different concentrations of TMAO. **d** Emission spectra of **B11** and **B11**‧‧‧TMAO at 77 K. **e** Energy transfer pathways for sensing TMAO by **B7**.

**B7** can interact directly with TMAO via the borono group to change the triplet energy level of the ligand and eventually change the energy transfer process of the system, resulting in the sensing property. To determine the function of the borono group, **B12** was synthesized with isophthalic acid, which has a molecular structure like that of H_2_BIPA but without the borono group (Supplementary data 4 and Supplementary Fig. 23). The emission intensities in the spectrum of **B12** at both 544 nm and 616 nm decreased slightly, and the value of *I*_616_/*I*_544_ did not change after adding TMAO (Supplementary Fig. 24), indicating that there was no direct interaction between TMAO and **B12**. In fact, **B7** has a one-dimensional channel with dimensions of 4.52 × 5.83 Å^2^, and the molecular size of TMAO is 2.95 × 3.51 × 4.16 Å^3^, indicating that TMAO can enter the channel of **B7**. The exposed borono group in the channel of **B7** is the interaction site to interact with TMAO in outer sphere via two modes: bonding or nonbonding interactions (Fig. 4a).

To further verify the interaction between the borono group and TMAO, ^1^H liquid and ^11^B solid-state MAS NMR experiments were performed. The ^1^H liquid NMR spectra of TMAO, H_2_BIPA, TMAO and H_2_BIPA mixture, and 5-hydroxy-isophthalic acid first exclude the possibility that H_2_BIPA is oxidized by TMAO to form 5-hydroxy-isophthalic acid in DMSO (Supplementary Fig. S25), suggesting that the borono group is stable in **B7**^45^. The ^11^B MAS NMR spectra of **B7** before and after treatment with TMAO are similar, and both are characterized by an asymmetric and featureless center band with intense spinning sidebands (Fig. 4b). The observed NMR line shape is induced by adjacent paramagnetic ions around ^11^B nuclei, i.e., the Tb^3+^ and Eu^3+^ within **B7** framework. The spectral width only slightly decreases after TMAO treatment, giving rise to a decrease of the quadrupolar coupling constant (*C*_Q_) from 4.4 MHz to 4.2 MHz. The change in ^11^B chemical shift value (*δ*_iso_) of −6 ppm clearly implies that **B7** can interact with TMAO via certain interaction^13^, and the small differences indicate such interaction is nonbonding (see the comparison between DFT-predicted *C*_Q_ and *δ*_iso_ of bonding and nonbonding modes in Supplementary Table 5, 6). In addition, after inclusion of TMAO by **B7**, differential FTIR spectra features attributed to B-O, B-O-H, and C-B were observed at 1312 cm^−1^, 1176 cm^−1^ and 1096 cm^−1^, respectively^46^ (Supplementary Fig. 26), which were attributed to the direct interaction of TMAO and **B7**.

The lifetimes of **B7** with TMAO were monitored at 544 and 616 nm (Supplementary Figs. 27, 28). The fluorescence lifetime at 544 nm decreased with the addition of TMAO, whereas the lifetime at 616 nm increased (Fig. 4c). The ratio of the lifetimes at 544 and 616 nm had a linear relationship with the concentration of TMAO (Supplementary Fig. 29 and Supplementary Table 7). This result indicates that the energy transfer process of **B7** changes by the interaction with TMAO via forming a **B7**···TMAO intermediate. The triplet energy levels (T_1_) of the antennas were measured using **B11** before and after interacting with TMAO (**B11**···TMAO) at 77 K, respectively (Fig. 4d). The energy of T_1_ of the antenna decreased from 22675 to 21231 cm^−1^ after combination with TMAO. The energy gap between T_1_ of the ligand and ^5^D_0_ of Eu^3+^ (17500 cm^−1^) changed from 5175 cm^−1^ to 3731 cm^−1^, which falls in the optimal energy gap range to excite Eu^3+^ (2500–4000 cm^−1^)^47^. The energy gap between the T_1_ of the ligand and ^5^D_4_ of Tb^3+^ (20500 cm^−1^) changed from 2175 cm^−1^ to 731 cm^−1^, which allows back-energy transfer^48^. These energy level changes led to an increase in Eu^3+^ emissions and a decrease in Tb^3+^ emissions (Fig. 4e). *X*-ray photoelectron spectroscopy (XPS) spectra of **B7** were also measured before and after immersion in TMAO (Supplementary Fig. 30). The B 1s peak of BIPA^2−^ was observed at 191.60 eV (+3 valence B) in **B7**, which shifted slightly to 191.45 eV after treatment with TMAO, indicating an interaction with TMAO and weak electron transfer from the O atom of TMAO to the B atom of BIPA^2−^ ^49,50^.

## Conclusions

A family of lanthanide metal-organic frameworks functionalized with borono groups were synthesized for the visual recognition of UV-vis silent molecules, demonstrated by TMAO that is the biomarker of cardiovascular disease. Well-matched interaction between the borono group of bilanthanide metal-organic framework and TMAO in the outer coordination sphere was successfully achieved, to provide a facile detection of TMAO. The superior sensitivity, selectivity and anti-interference ability of this borono-functionalized lanthanide metal-organic framework is based on the inverse variation trend of the emission intensities of two emission centers, which is originated from TMAO-influenced energy transfer process. This work not only provides a facile strategy for TMAO on-site detection that could be used to reduce the mortality rate of cardiovascular diseases but also reveal the fundamentals in molecular level for the design of advanced sensing materials for UV-vis silent molecules that related to public health and green environments.

## Methods

### Synthesis

H_2_BIPA (1 mmol, 0.208 g), 10 mL EG, 10 mL DMA and 40 mL H_2_O were added to a 100 mL beaker to form a solution under ultrasound. 4 mL as prepared solution and 1 mL terbium acetate tetrahydrate aqueous solution (0.1 M) were added to a 10 mL glass vial, which was then sealed and heated at 90 °C for 12 h. The obtained crystals (**B1**) were washed with distilled water for three times and then dried in air. **B2**-**B11** were synthesized similarly to **B1**, except 0.1 M terbium/europium/gadolinium acetate aqueous solution with different volumes (1 mL in total) were used. **B12** was synthesized similarly to **B7**, except changing H_2_BIPA to H_2_IPA. Isomorphic **B12-Tb** was synthesized similarly to **B12**, except using 1 mL 0.1 M terbium acetate aqueous solution. Elemental analysis (EA), inductively coupled plasma-atomic emission spectrometry (ICP-AES) results and yields of all compounds were summarized in Supplementary Table 8.

### Characterization

The single-crystal data were collected using a Rigaku SuperNova or a Rigaku XtaLAB Mini II single-crystal diffractometer equipped with graphite-monochromatic Mo-*K*α radiation (λ = 0.71073 Å). The structures were solved by SHELXS (direct methods) and refined by SHELXL (full matrix least-squares techniques) in the *Olex*2 package^51,52^. PXRD measurements were performed using a Rigaku Smartlab SE *X*-ray diffractometer equipped with a Cu-tube and a graphite monochromator scanning over the range of 5–50° at the scan rate of 0.2° s^−1^ at room temperature. Simulations of the PXRD patterns were carried out with the single-crystal data and diffraction crystal module of the Mercury program available free of charge via http://www.ccdc.cam.ac.uk/mercury/. The SEM images and EDS elemental mapping were obtained using Hitachi SU3500 scanning electron microscopy equipped with Brooke energy spectrometer. The FTIR spectroscopy were carried out on a Bruker ALPHA spectrophotometer. TGA data were obtained on TGA 2 STAR^e^ System of METTLER TOLEDO under nitrogen atmosphere with the heating rate of 10 °C min^−1^. EA for C, H and N were carried out using a Vario EL cube elemental analyzer. ICP-AES analyses were conducted using a Thermo IRIS Advantage instrument. UV-vis absorption spectra were measured with a SHIMADZU UV-2600 spectrophotometer. XPS were acquired using PHI5000Versa probe equipped ESCALAB 250xi. ^1^H NMR spectra were recorded on a Bruker AV400 spectrometer. The solid-state MAS ^11^B NMR experiments were performed on a 400 MHz Digital Solid-State NMR Spectrometer with 2.5 mm MAS probe at 20 kHz. Luminescence spectra were recorded on an Edinburgh FS5 fluorescence spectrophotometer equipped with a xenon lamp and pulsed flash lamps at room temperature. Photos were taken by an iPhone 12 and chroma values (*R*, *G* and *B*) of every photo are acquired using *Swatches* app obtained from *App Store*.

### DFT calculation

The ground-state structure optimization, the highest occupied molecular orbital (HOMO) and the lowest unoccupied molecular orbital (LUMO) energy levels of H_2_BIPA, TMAO and H_2_BIPA···TMAO were calculated by the DFT method at B3LYP/6-31 G* level by the Gaussian 09 W program package^53^. Molecular modeling simulation of **B1**···TMAO was performed at ultrafine level in Forcite module using Materials Studio. The energy and the force were set as 2.0×10^−5 ^kcal/mol and 0.001 kcal/mol/Å, respectively. The displacement was 1.0 ×10^−5 ^Å.

### Luminescence measurements

The finely grounded sample (30 mg) was dispersed into distilled water (100 mL) to form aqueous dispersions. The mixture was sonicated for 10 min. The luminescence spectra of **B2-B9** dispersions upon excitation at 254 nm were measured in situ after incremental addition of freshly prepared water solution containing 100 mM TMAO. Interference experiments were performed using 2 mL aqueous dispersions of **B7**. Fluorescent lifetimes of **B7** without or with TMAO with different concentrations were obtained upon excitation at 254 nm. For the smartphone application, after adding 100 mM TMAO with different volumes to 0.5 mL aqueous dispersions of **B7**, respectively, images were taken by an iPhone 12.

## Supplementary information

Supplementary Material

## Data Availability

All the data of this study are available. The authors declare that the data supporting the findings of this study are available within the article and its Supplementary Information files. Supplementary Data 1-4 are *X*-ray crystallographic data for **B1**, **B10**, **B11** and **B12-Tb**, which have been deposited at the Cambridge Crystallographic Data Centre (CCDC), under deposition numbers 2092326 - 2092329, respectively. These data can also be obtained free of charge from the CCDC via www.ccdc.cam.ac.uk/data_request/cif. The data that support the findings of this study are available from the corresponding author.
