# Supplementary material for: Detection of the UV-vis silent biomarker trimethylamine-N-oxide via outer-sphere interactions in a lanthanide metal-organic framework
Source: Commun Chem. 2022 Jun 22;5:74. doi: 10.1038/s42004-022-00690-8 (PMC9814541; doi:10.1038/s42004-022-00690-8)
Supplement: Supplementary file 1 — Supplementary Material [file 42004_2022_690_MOESM1_ESM.pdf]

Supplementary Information for

Detection of the UV-vis silent biomarker trimethylamine-*N*-oxide via outer-sphere interactions in a lanthanide metal-organic framework

Min et al.

Contents

1. Design strategy for TMAO detection S2

2. Structure and characterizations S3-S5

3. Luminescent properties S6-S7

4. Detection of TMAO S8-S10

5. Limit of detection S11

6. Smartphone application for TMAO detection S11

7. Mechanism study S12-S17

8. Supplementary Tables S18-S22

9. Reference S23

1. Design strategy for TMAO detection

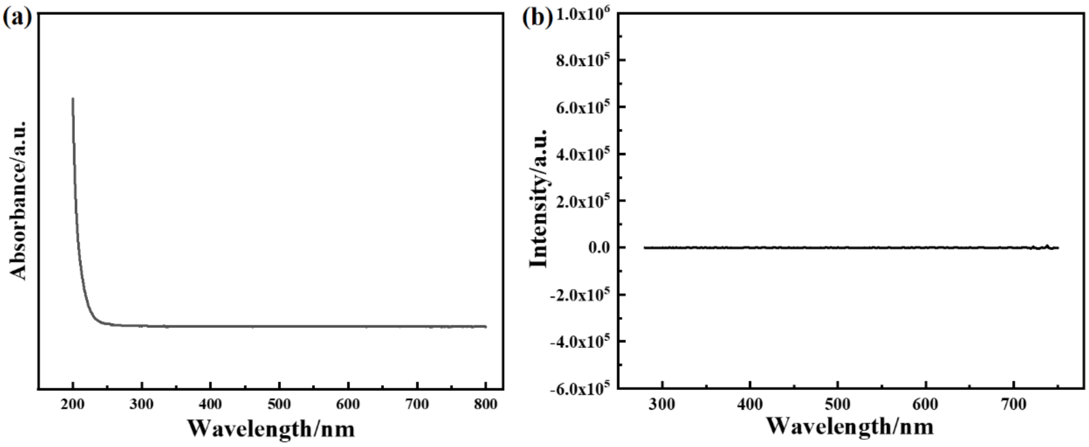

Supplementary Figure 1. The UV-vis absorption spectrum (a) and emission spectrum excited at 254 nm (b) of 25 mM TMAO aqueous solution.

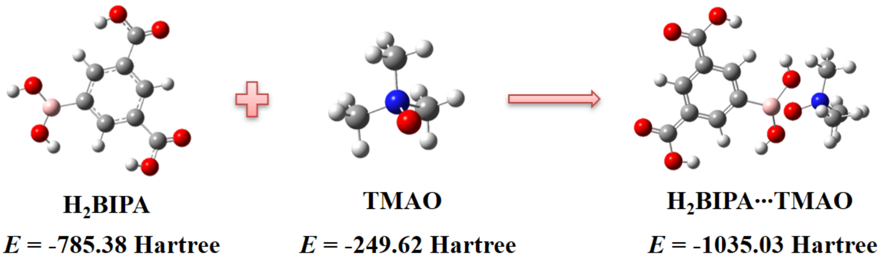

Supplementary Figure 2. The optimized ground-state structures of H_2BIPA, TMAO and H_2BIPA···TMAO intermediate calculated by DFT method at the B3LYP/6-31G* level.

2 Structure and characterizations

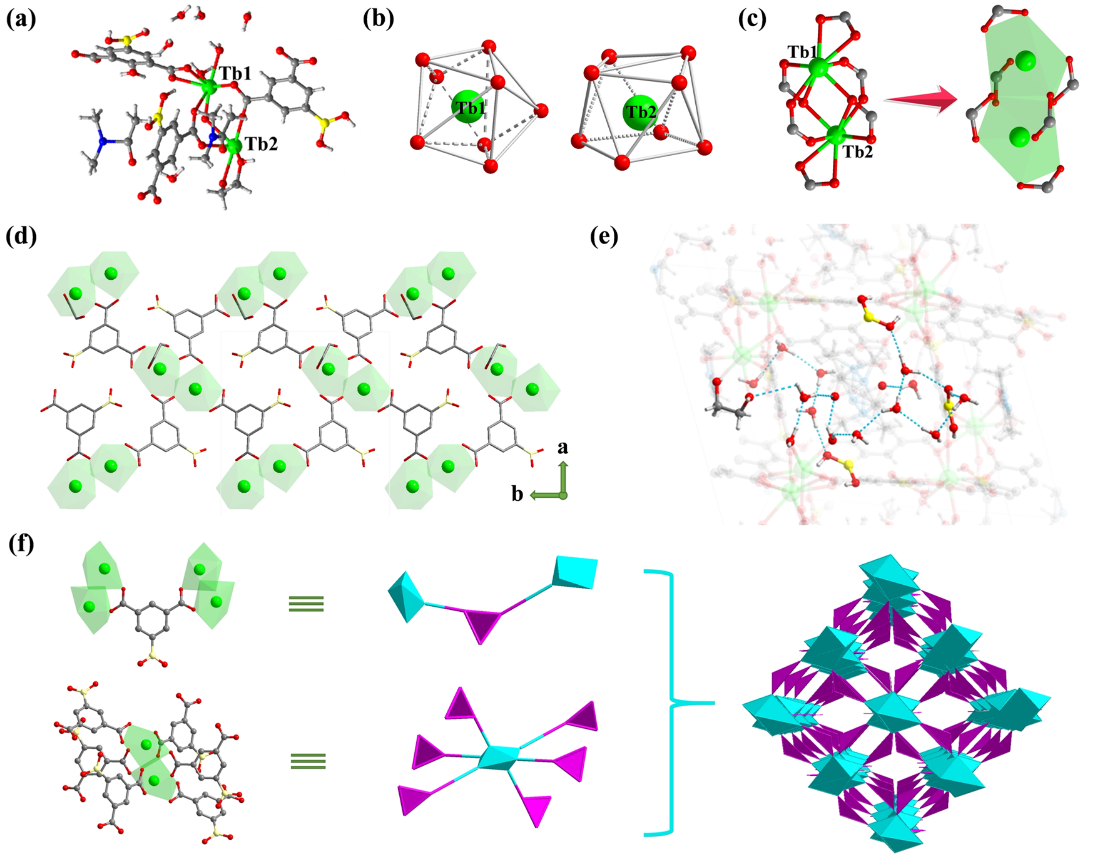

Supplementary Figure 3. (a) the asymmetric unit of **B1**; (b) the coordination environments of Tb^{3+} in **B1**; (c) the binuclear unit of **B1**; (d) 2D network of **B1** in the *ab* plane; (e) hydrogen bond networks in **B1**; (f) Topological analysis of **B1**. Atom codes: Tb (green), C (gray), B (yellow) and O (red), Hydrogen atoms and solvent molecules are omitted for clarity.

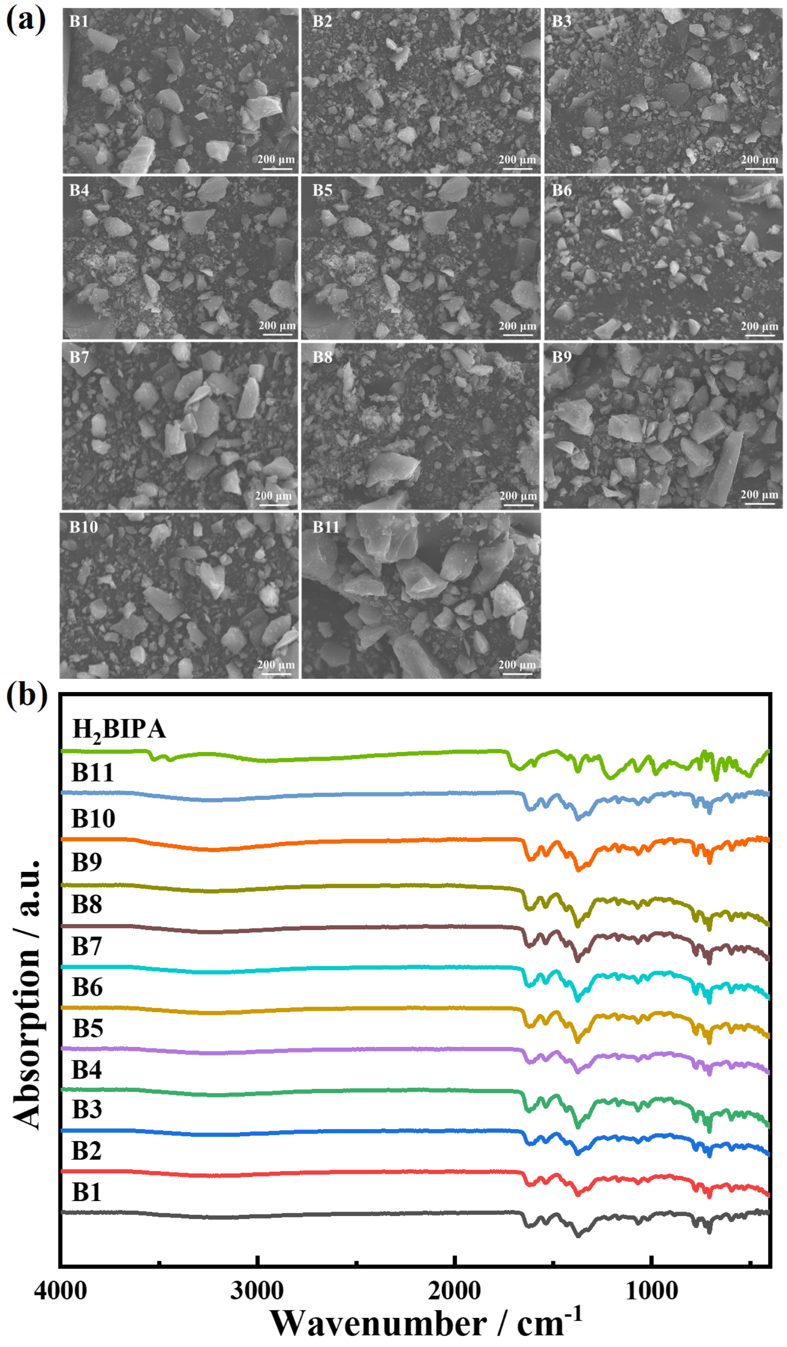

Supplementary Figure 4. (a) SEM images of **B1-B11**; (b) FTIR of H_2BIPA and **B1-B11.**

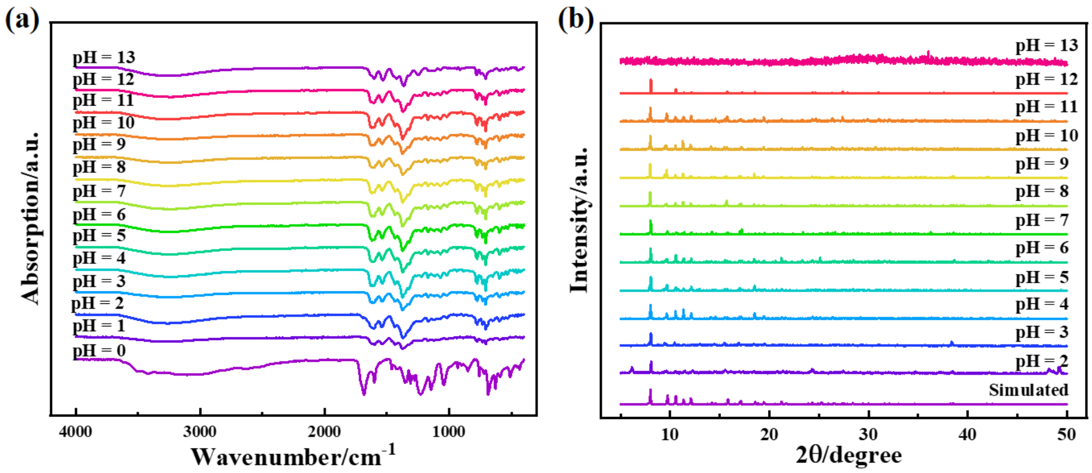

Supplementary Figure 5. FTIR (a) and PXRD patterns (b) of **B1** after immersing in water with different pH values for 24 hours.

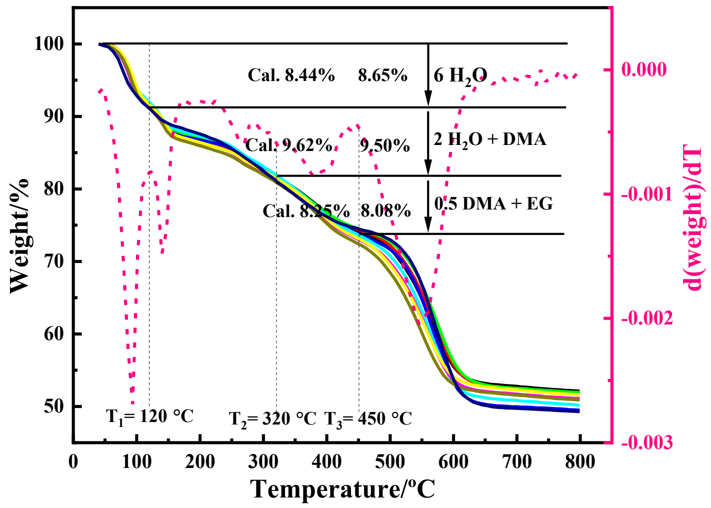

Supplementary Figure 6. TGA curves of **B1**-**B11**.

3. Luminescent properties

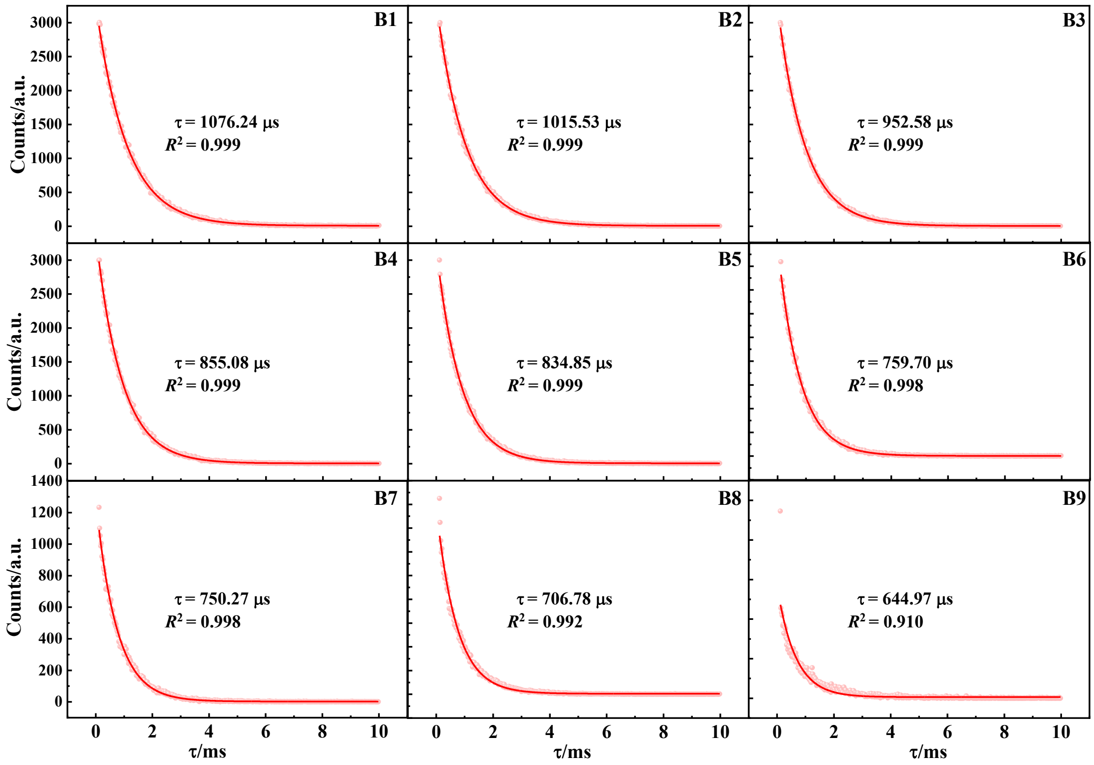

Supplementary Figure 7. The lifetimes at 544 nm of **B1**-**B9** excited at 254 nm.

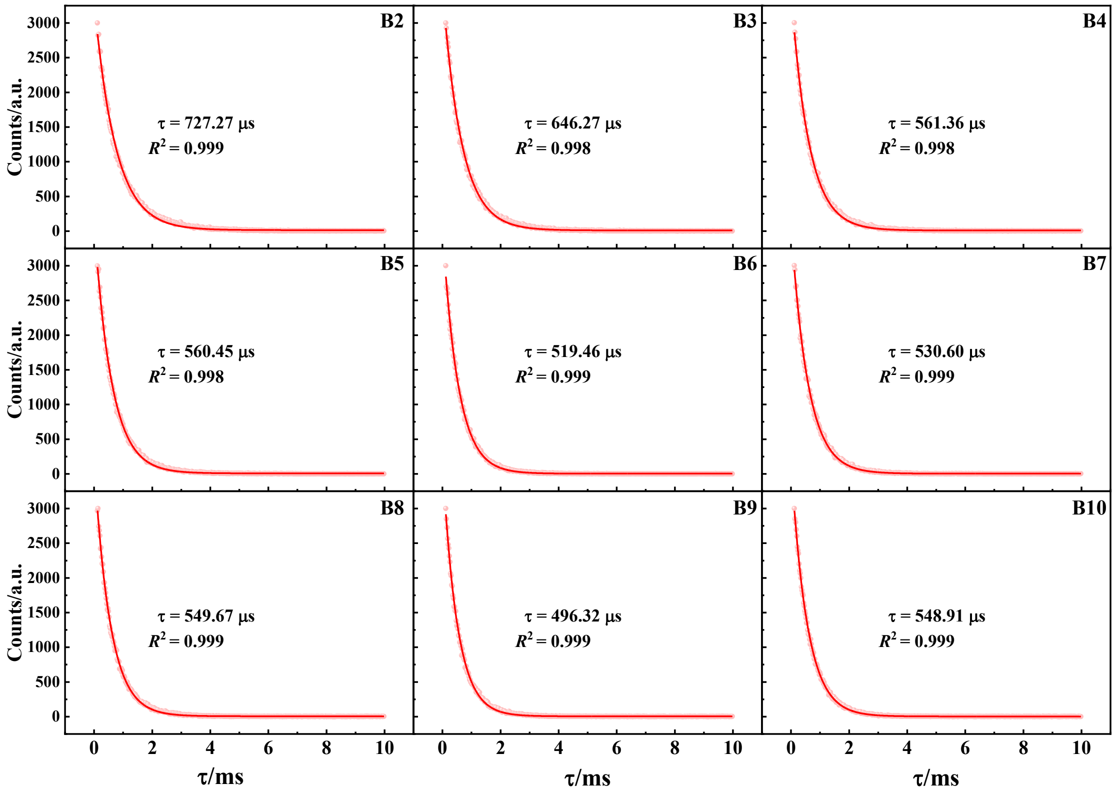

Supplementary Figure 8. The lifetimes at 616 nm of **B2**-**B10** excited at 254 nm.

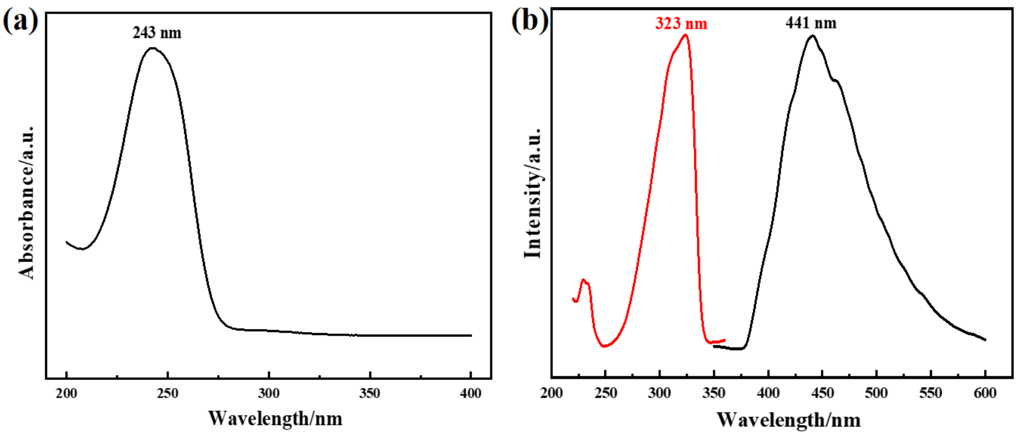

Supplementary Figure 9. (a) The UV spectrum of H_2BIPA; (b) The excitation (red) and emission (black) phosphorescence spectrum of **B11** at 77 K.

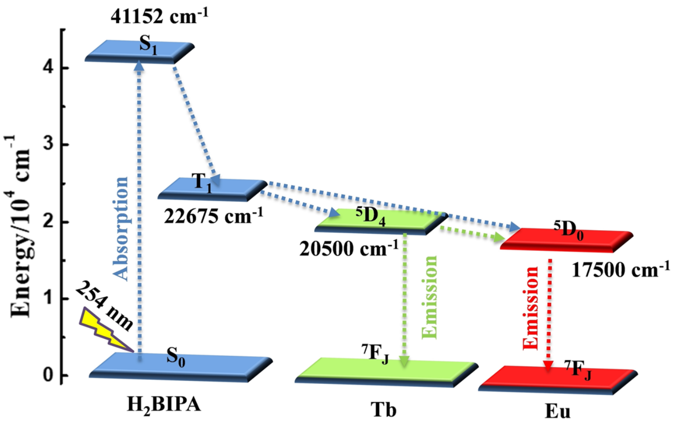

Supplementary Figure 10. The energy transfer process of **B1**-**B10**.

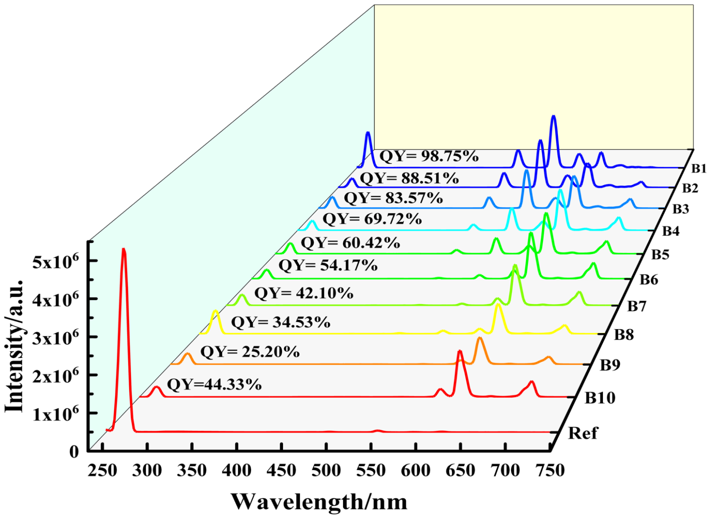

Supplementary Figue 11. The solid-state luminescent quantum yields of **B1-B10**.

4. Detection of TMAO

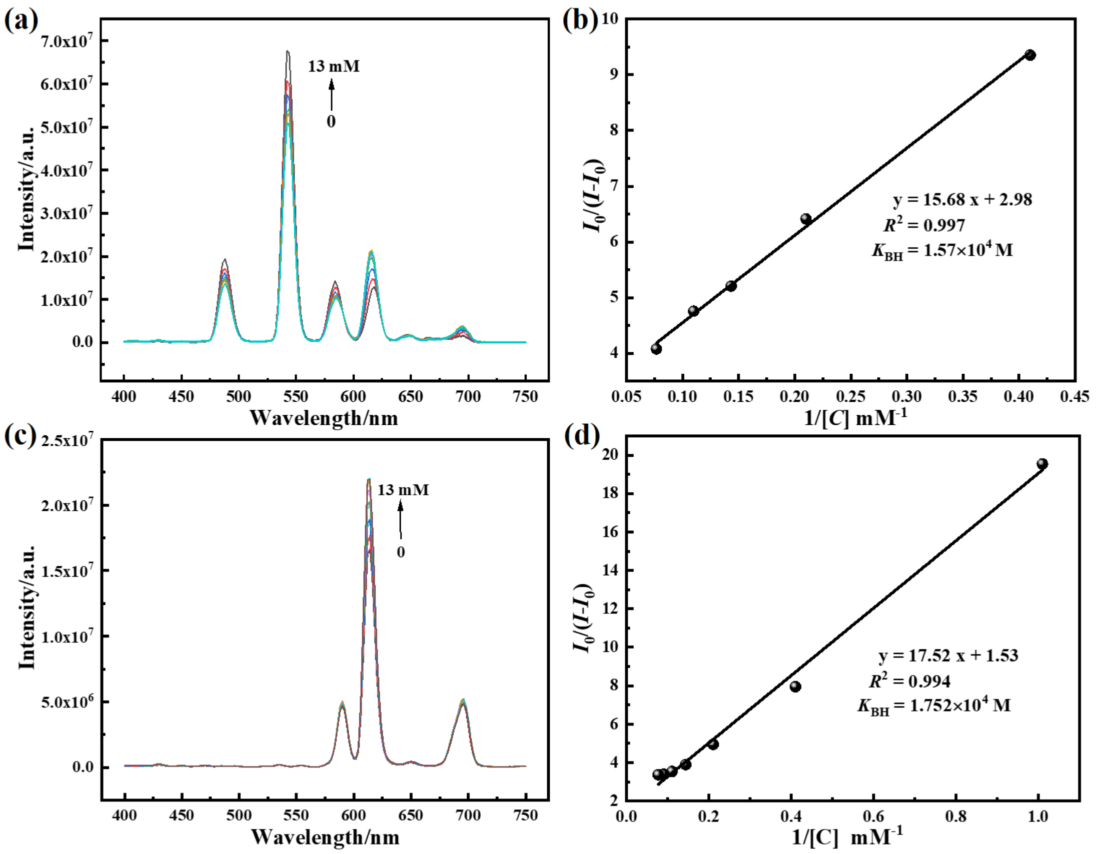

Supplementary Figure 12. Fluorescence responses of (a) **B1** and (c) **B10** aqueous dispersions towards TMAO; Benesi–Hildebrand curves of **B1** at 544 nm (b) and **B10** at 616 nm (d), and the solid lines are fitting results.

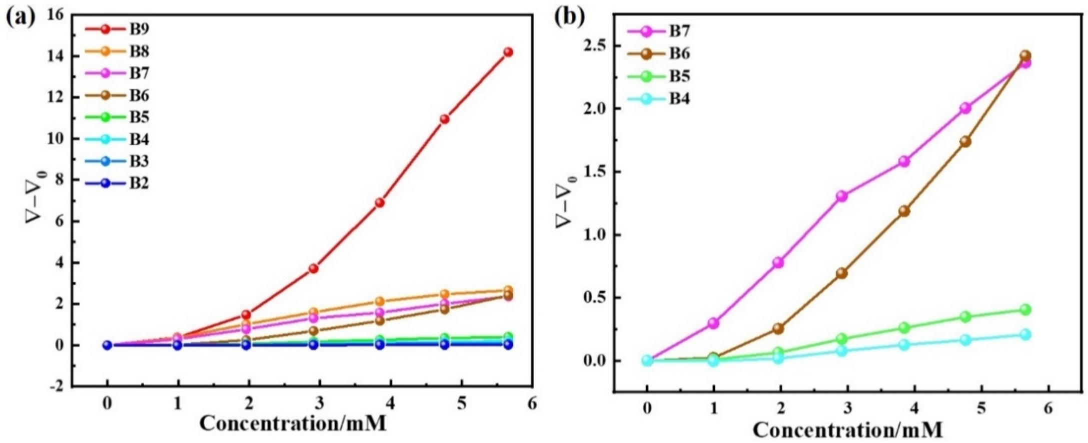

Supplementary Figure 13. (a) Fluorescence responses of **B2** - **B9** towards TMAO with different concentration (∇ = I_{616}/I_{544}); (b) Comparison of **B4** - **B7** towards TMAO with different concentrations.

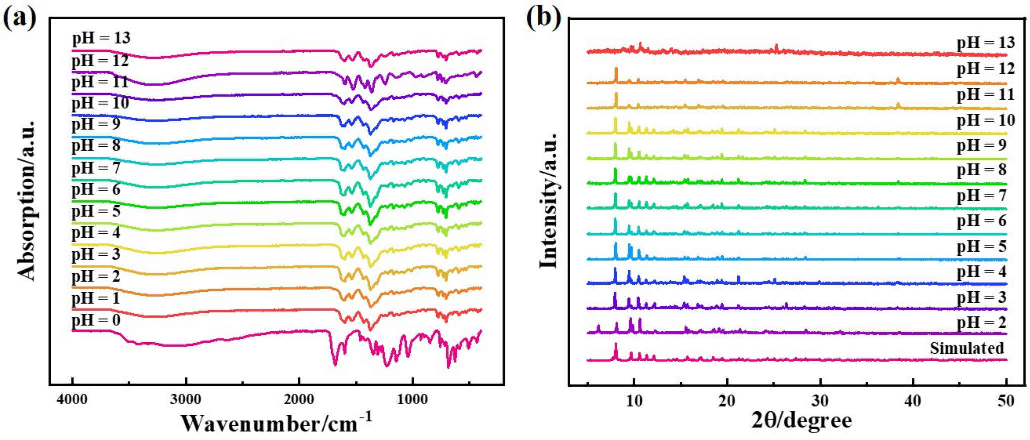

Supplementary Figure 14. FTIR (a) and PXRD patterns (b) of **B7** after immersing in water with different pH values for 24 hours.

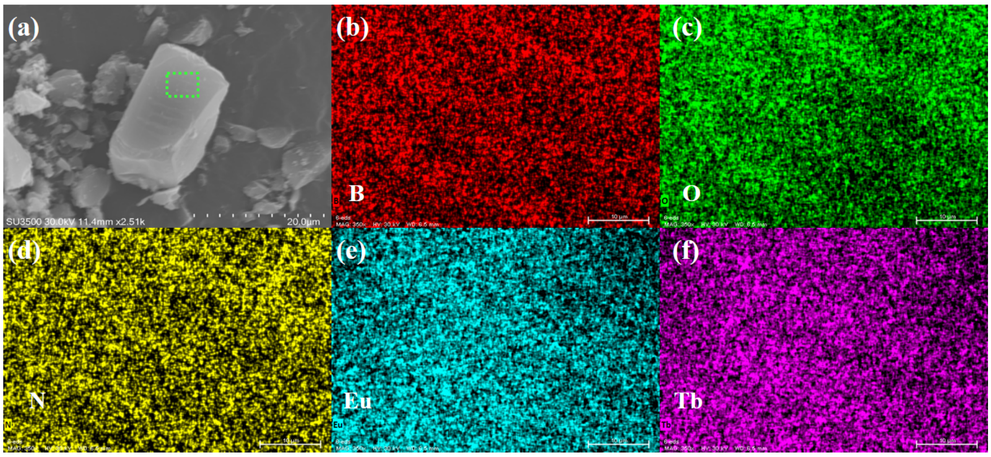

Supplementary Figure 15. the SEM of B7 (a); EDS elemental mapping of B (b), O (c), N (d), Eu (e), Tb (f).

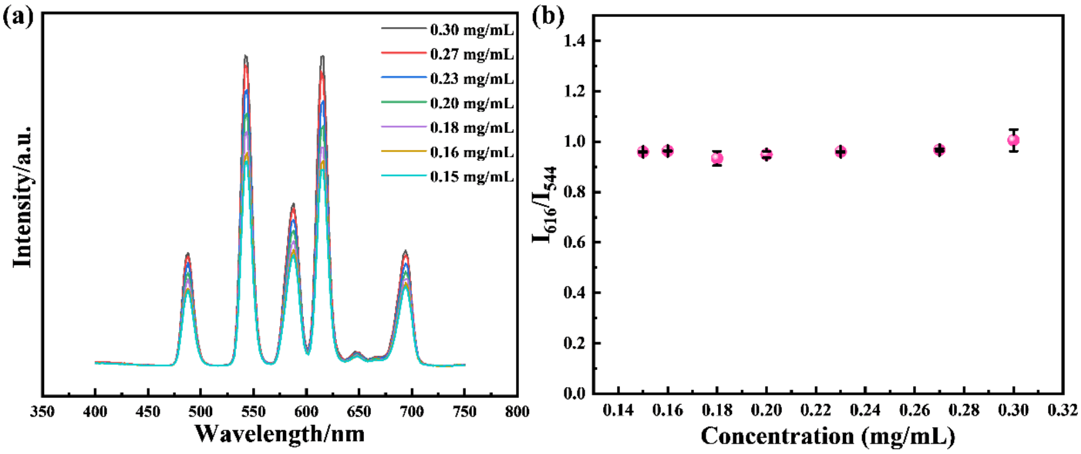

Supplementary Figure 16. (a) Fluorescence spectra of **B7** aqueous suspension with different concentrations (0.15 - 0.30 mg/mL); (b) I_{616}/I_{544} of **B7** aqueous suspension with different concentrations. Error bar shows the standard deviation of test results.

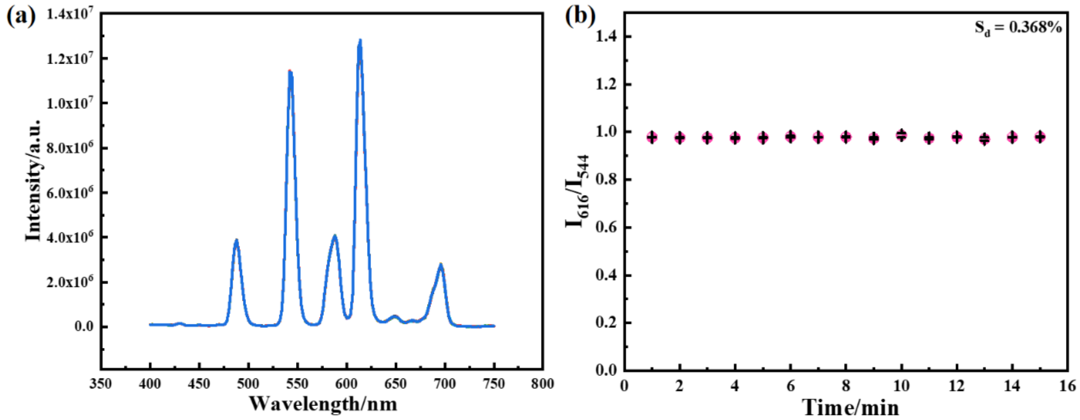

Supplementary Figure 17. (a) Time-dependent luminescent spectra of **B7** for fifteen times; (b) Luminescent intensities of **B7**. S_d inset is the standard deviation of replicate luminescent intensities of blank solutions for fifteen times. Error bar shows the standard deviation of test results.

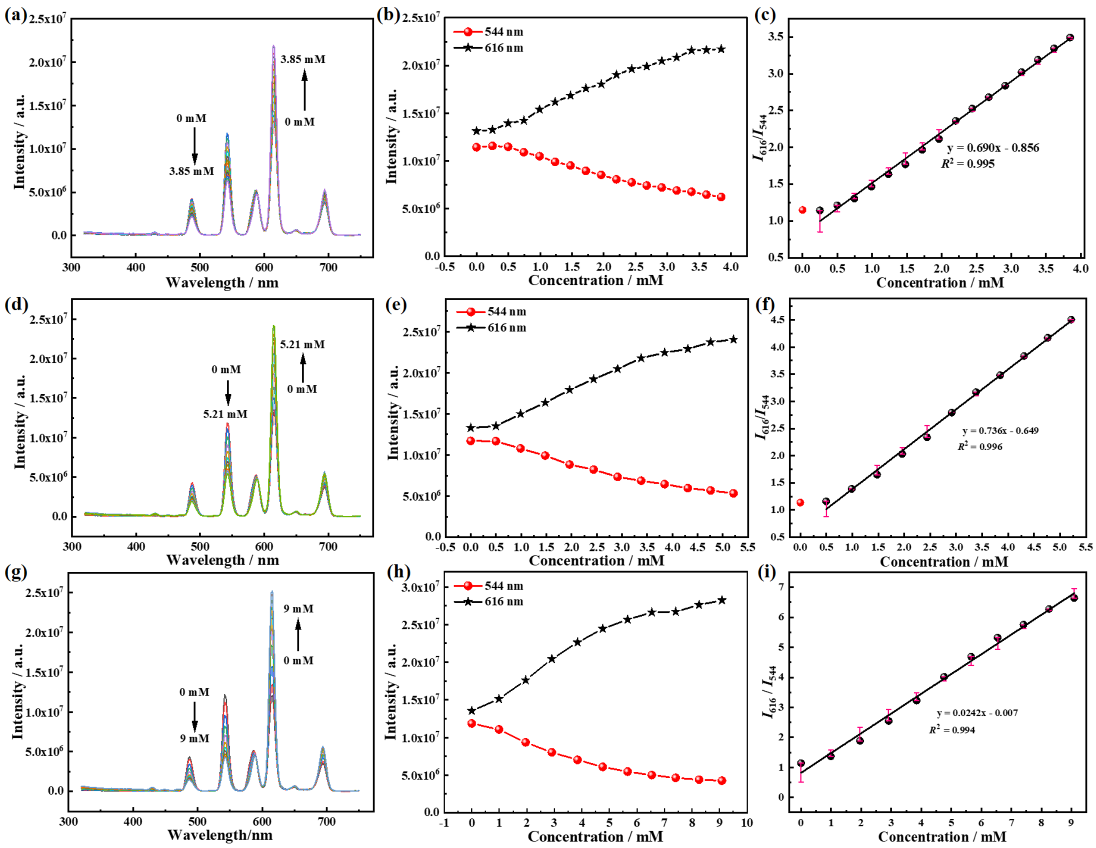

Supplementary Figure 18. Three independent fluorescence titrations with incrementally adding 5 μL (a - c), 10 μL (d - f) and 15 μL (g - i) 100 mM TMAO to **B7** aqueous dispersions. Error bar shows the deviation of the test results and theoretical values.

5. Limit of detection

Supplementary Discussion: The limit of detection (LOD) was calculated according to the 3σ IUPAC criteria using the following equations:[S1]

$$S_d = \sqrt{\frac{1}{N-1}\sum_{i=1}^{N}(V_i - V_{av})^2} \quad (i = 1, 2, 3, \ldots, N)$$

$$\mathrm{LOD} = \frac{3S_d}{\mathrm{Slope}}$$

where S_d is the standard deviation of replicate luminescent intensities of blank solutions for fifteen times; V_i are ratios of luminescence intensities at 616 nm and 544 nm of **B7** aqueous dispersions in the absence of TMAO; V_{av} is the average of V_i $(\frac{I_i}{I_0})$. Slope is obtained from Figure 2c. The LOD was calculated as 15.6 μM.

6. Smartphone application for TMAO detection.

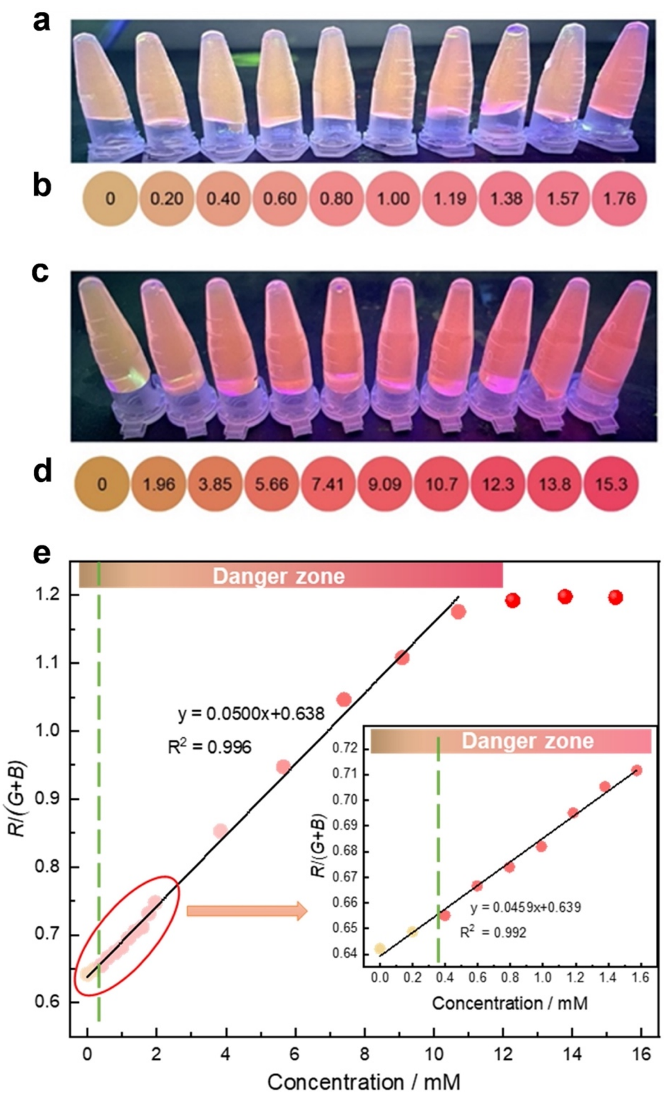

Supplementary Figure 19. Color (a) and chromaticity (b) changes of **B7** with different concentrations of TMAO (0 to 1.76 mM). Color (c) and chromaticity (d) changes of **B7** with different concentrations of TMAO (0 to 15.3 mM). (e) *R/(G+B)* value versus the concentration of TMAO. The inset shows a magnified view of low concentrations of TMAO.

7. Mechanism study

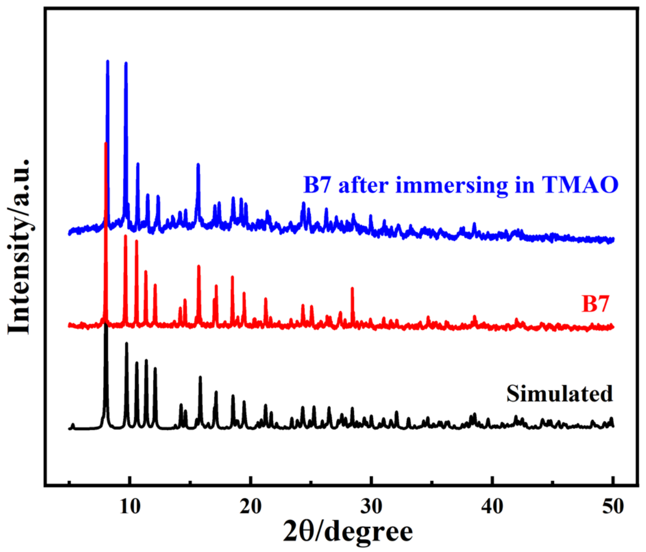

Supplementary Figure 20. The PXRD of **B7** before and after immersing in 10 mM TMAO for 12 h.

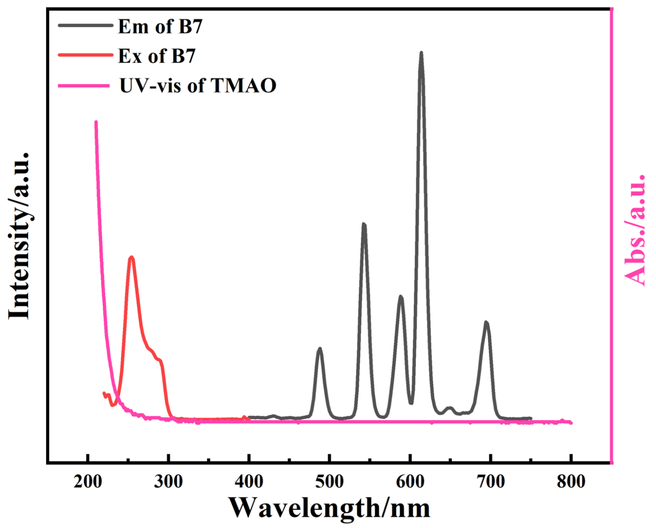

Supplementary Figure 21. The excitation spectrum (Ex, red), emission spectrum (Em, black) of **B7** and the UV-vis spectrum (pink) of 25 mM TMAO.

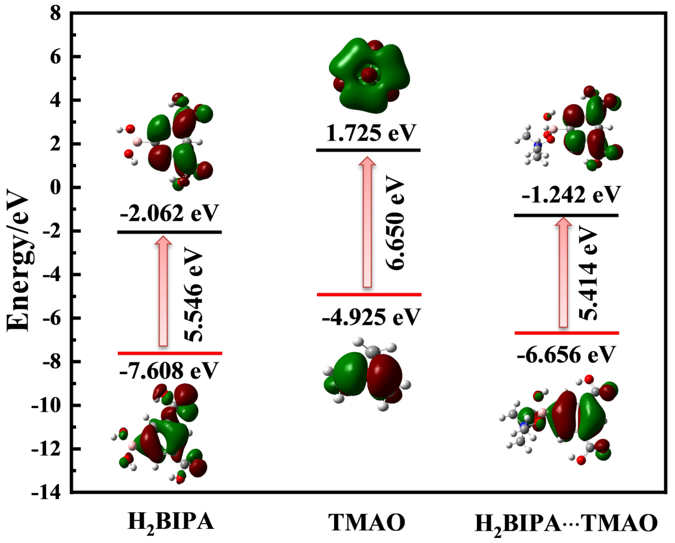

Supplementary Figure 22. HOMO and LUMO energy levels for H_2BIPA, TMAO and H_2BIPA···TMAO.

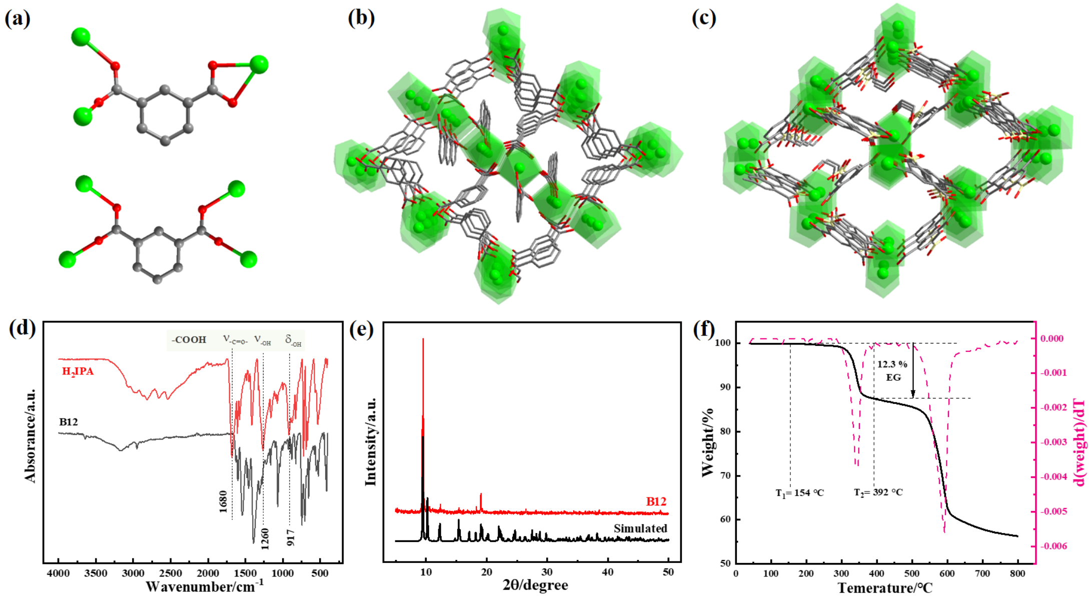

Supplementary Figure 23. (a) The coordination environment of IPA^{2-} in **B12-Tb**; 3D framework of **B12-Tb** (b) and **B1** (c); (d) FTIR spectra of H_2IPA and **B12**; (e) The PXRD of **B12** and the simulated one obtained from **B12-Tb**; (f) The TGA curve of **B12**. Atom codes: Tb (green), C (gray) and O (red). Hydrogen atoms are omitted for clarity.

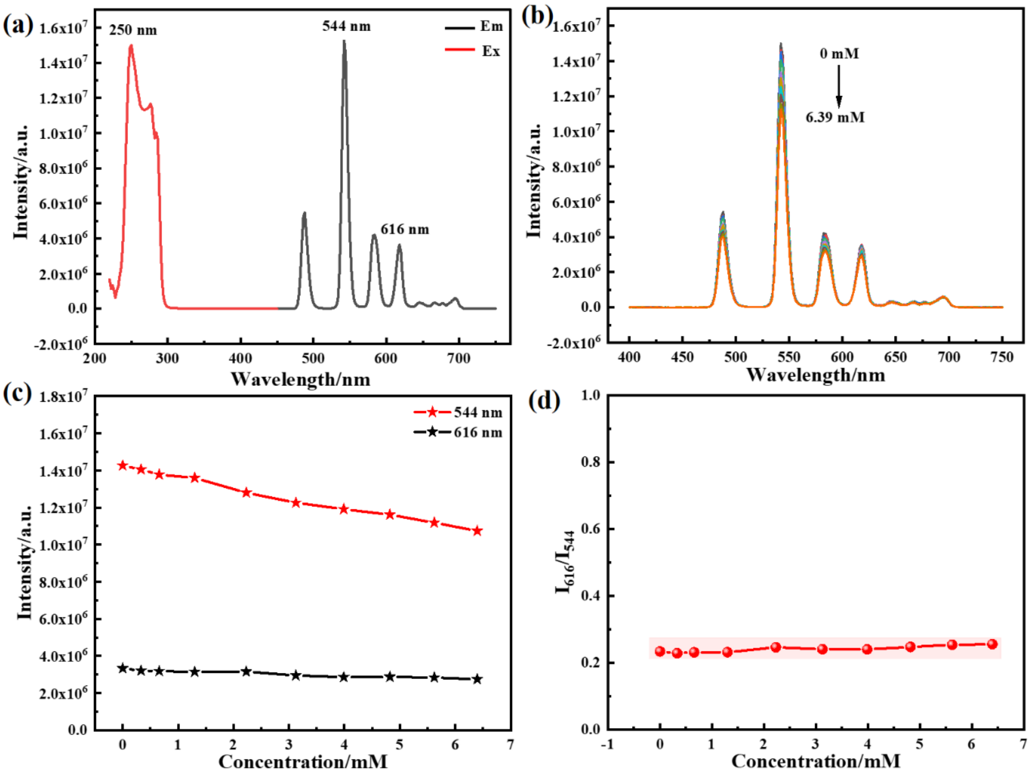

Supplementary Figure 24. (a) The excitation spectrum (Ex, red) and emission spectrum (Em, black) of **B12**; (b) The fluorescence response of **B12** toward TMAO with different concentration; (c) Fluorescence intensity at 544 nm and 616 nm of **B12** varies with the concentration of TMAO; (d) Ratio of fluorescence intensity at 616 nm and 544 nm of **B12** varies with the concentration of TMAO.

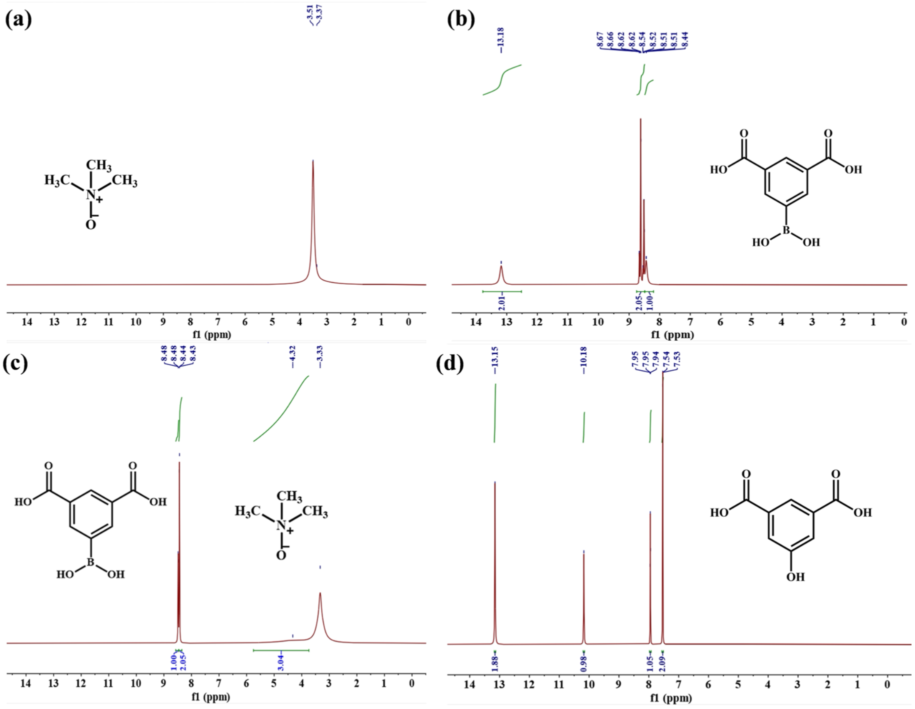

Supplementary Figure 25. ^{1}H liquid NMR spectra of (a) 3 mg TMAO in 0.5 mL DMSO-d_6; (b) 3 mg H_2BIPA in 0.5 mL DMSO-d_6; (c) 3 mg TMAO and 3 mg H_2BIPA in 0.5 mL DMSO-d_6; (d) 3 mg 5-hydroxy-isophthalic acid in 0.5 mL DMSO-d_6. Part solvent peaks are removed using MestReNova software for clarity.

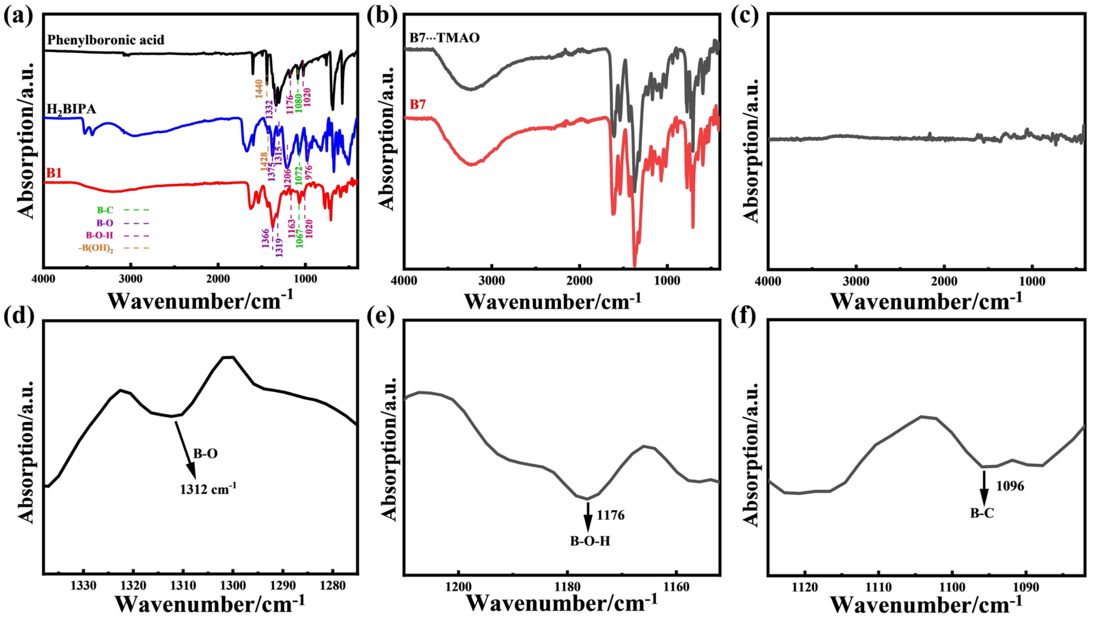

Supplementary Figure 26. (a) FTIR of phenylboronic acid, H_2BIPA and **B1**; (b) FTIR of **B7** and **B7**····TMAO; (c) Normalized differential FTIR of **B7** and **B7**····TMAO; The localization normalized differential FTIR for (d) B-O bond, (e) B-O-H bonds and (f) B-C bond.

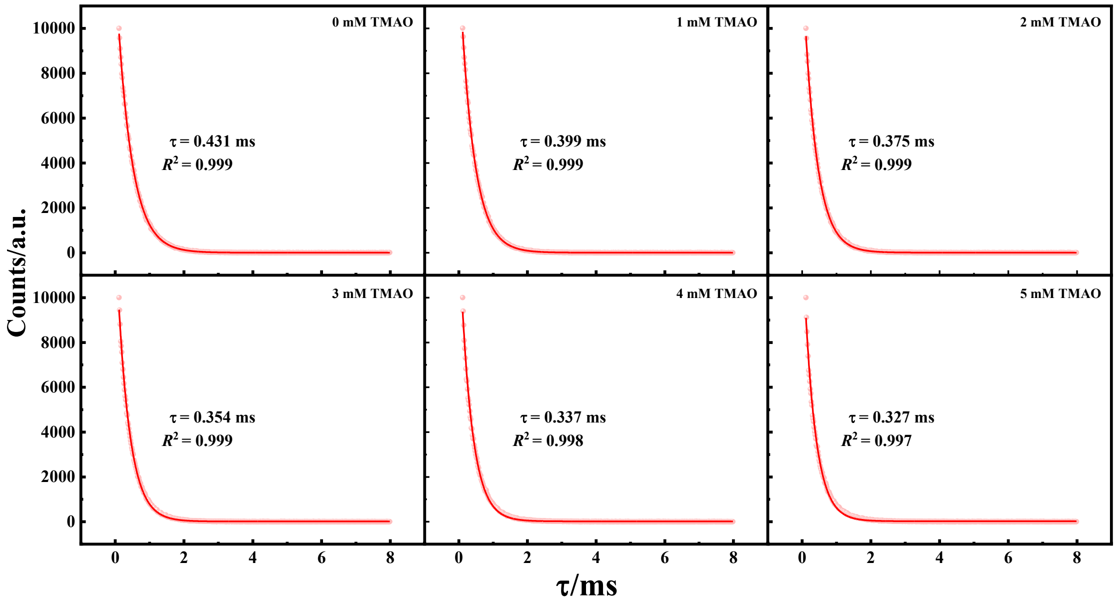

Supplementary Figure 27. The lifetimes at 544 nm of **B7** with TMAO excited by 254 nm.

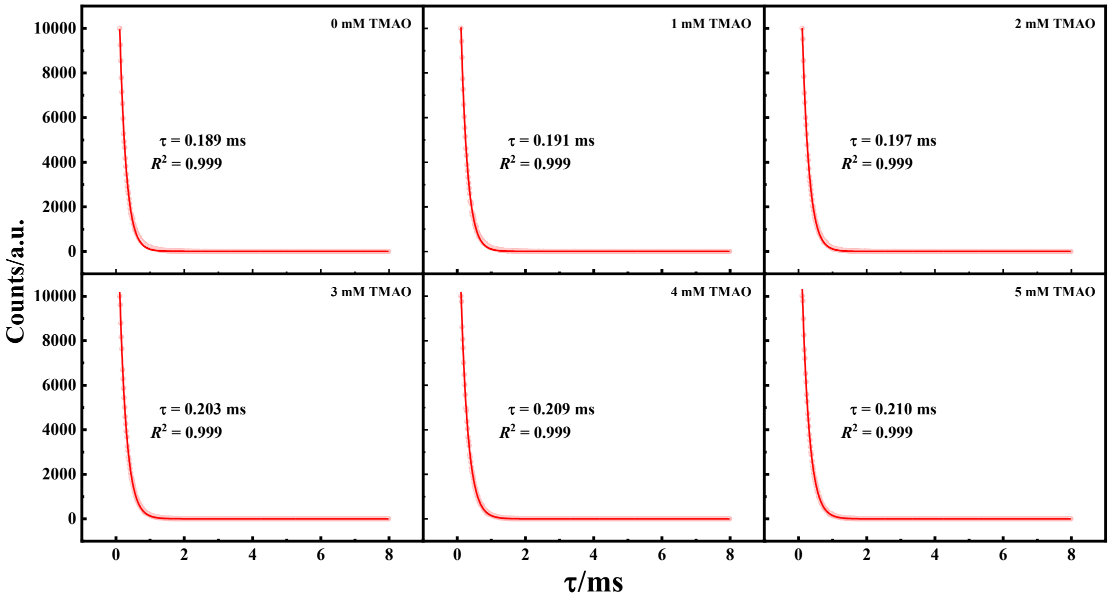

Supplementary Figure 28. The lifetimes at 616 nm of **B7** with TMAO excited by 254 nm.

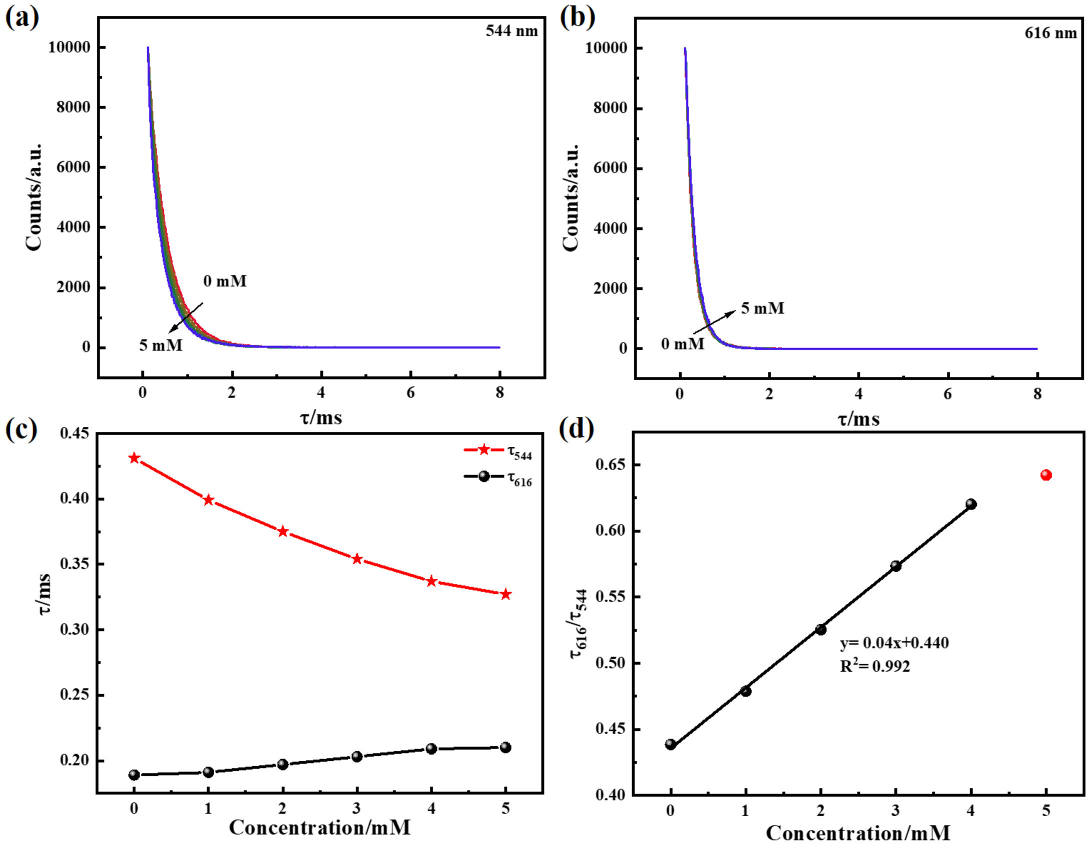

Supplementary Figure 29. Change of fluorescence lifetime curves at 544 nm (a) and 616 nm (b) of **B7** water dispersions treating with TMAO of different concentration excited by 254 nm; Fluorescence lifetimes at 616 nm and 544 nm (c) and their ratio (d) of **B7** water dispersion varies with the concentration of TMAO.

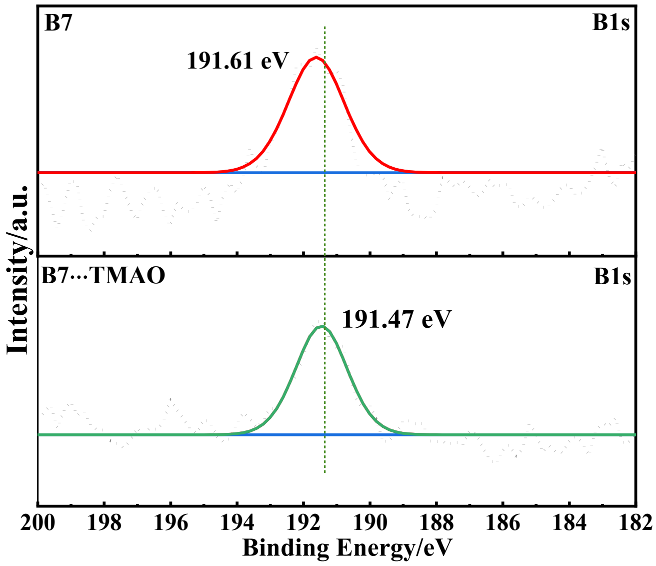

Supplementary Figure 30. XPS of **B7** and **B7** after immersing in 10 mM TMAO (**B7···TMAO**) for 8h.

8. Supplementary Tables

Supplementary Table 1. Selected materials for UV-silent biomarkers of different diseases.

Diseases	Materials	Methods	Biomarkers	Fluid	LR (μM)	LOD (μM)	Ref.
CVD	**B7**	Fluorescent	TMAO	-	0-9000	15.6	This work
CVD	-	UHPLC-MS/MS	TMAO	Urine	13.3-2000	-	S2
CVD	-	GC-MS	TMAO	Urine	14.9-956	14.9	S3
CVD	MIP/ITO	Electrochemical	TMAO	Urine	13.3-199	20.0	S4
CVD	GMD	Fluorescent	TMAO	Urine	0-1.22	28.88	S5
CVD	PSM	Colorimetry	TMAO	Urine	10-750	~4	S6
OC	Ln-ZMOFs	Fluorescent	LPA	Serum	1.4-43.3	-	S7
OC	PDAs	Fluorescent	LPA	Serum	0.5–8	0.5	S8
Cancer	Dye@MMT	Fluorescent	Spermine<br>Spermidine	Urine	0-27<br>0-45	-	S9
LC	NvBNs	Electrochemical	Heptanal	Plasma	-	10-8	S10
Diabetes	Ni-MOF	Electrochemical	Glucose	Serum	2-2000	0.1	S11
Argininemia	Eu-L_1	Fluorescent	Arginine	-	0-160	0.015	S12
PC	IPN	Electrochemical	Sarcosine	Urine	5-1100	0.38	S13

LR: Linear range; LOD: limit of detection; Ref.: Reference; CVD: Cardiovascular disease; **B7**: $\{[Tb_{0.85}Eu_{1.15}(BIPA)_3(EG)(H_2O)_2]\cdot 1.5DMA\cdot 6H_2O\}_n$ (H_2BIPA = 5-boronoisophthalic acid, EG = ethylene glycol, DMA= *N*, *N*-Dimethylacetamide); TMAO: Trimethylamine-*N*-Oxide; UHPLC-MS/MS: ultra-high performance liquid chromatography–tandem mass spectrometry; GC-MS: gas chromatography–mass spectrometry; MIP: molecularly imprinted polymer; ITO: indium tin oxide; GMD: Guanidinium-Modified Calixarene; PSM: Porous Silica-Dye Microspheres; OC: Ovarian Cancer; Ln-ZMOFs: $[(Eu_xTb_{1-x})_{48}(NO_3)_{48}(bpdc)_{48}]\cdot G_x$ (G = guest, H_2bpdc = 2,2′ bipyridine-6,6′ -dicarboxylic acid); LPA: Lysophosphatidic acid; PDAs: polydiacetylenes; Dye: coumarin; MMT: montmorillonite; LC: lung cancer; NvBNs: nanovesicle-based bioelectronic nose; Ni-MOF: $[Mn_2\{Ni(C_2S_2(C_6H_4COO)_2)_2\}(H_2O)_2]\cdot 2DMF$; L_1: tetrakis(4-carboxyphenyl)pyrazine; PC: Prostate Cancer; IPN: imprinted polymeric nanobeads.

Supplementary Table 2. The crystal data and structural refinement parameters for **B1**, **B10**, **B11** and **B12-Tb**.

Compounds	**B1**	**B10**	**B11**	**B12-Tb**
Formula	$C_{64}H_{101}B_6N_3O_{59}Tb_4$	$C_{64}H_{101}B_6N_3O_{59}Eu_4$	$C_{64}H_{101}B_6N_3O_{59}Gd_4$	$C_{14}H_{12}O_8Tb$
Formula weight	2557.01	2529.17	2550.33	467.16
λ / Å	0.71073	0.71073	0.71073	0.71073
Crystal system	Monoclinic	Monoclinic	Monoclinic	Monoclinic
Space group	$P2_1/c$	$P2_1/c$	$P2_1/c$	$C2/c$
a / Å	17.4981(4)	17.4824(5)	17.4468(2)	10.8191(8)
b / Å	14.6568(3)	14.6916(4)	14.6288(2)	15.6295(10)
c / Å	19.1640(4)	19.1570(7)	19.1050(2)	19.3900(14)
α / deg	90	90	90	90
β / deg	106.552(2)	106.671(3)	106.901(2)	105.348(7)
γ / deg	90	90	90	90
V / Å^3	4711.25(18)	4713.6(3)	4665.48(11)	3161.9(4)
Z	2	2	2	8
ϱ_{calc} / g mm^{-3}	1.803	1.782	1.815	1.963
μ / mm^{-1}	3.074	2.733	2.916	4.511
Reflections collected	31773	41305	19570	14418
Independent reflections	9761	9759	8206	3267
R(int)	0.0282	0.0355	0.0440	0.0395
2θ range / deg	3.932 - 52.998	3.928 - 53.000	5.67 - 50.000	4.356 - 52.972
F(000)	2536.0	2520.0	2528.0	1800.0
GOF on F^2	1.059	1.043	1.033	1.081
$^aR_1$ / $^bwR_2$ [I > 2σ (I)]	0.0294/0.0693	0.0302/0.0682	0.0384/0.0897	0.0247/0.0612
$^aR_1$ / $^bwR_2$ (all data)	0.0408/0.0727	0.0430/0.0719	0.0503/0.0993	0.0286/0.0625
Largest diff. peak / hole / e Å^{-3}	1.07/-0.69	1.26/-0.71	1.91/-0.94	0.80/-0.98

$^aR_1 = \sum||F_0| - |F_c||/\sum|F_0|$; $^bwR_2 = [\sum w(F_0^2 - F_c^2)^2/\sum w(F_0^2)^2]^{1/2}$.

Supplementary Table 3. The $^5D_4$ and/or $^5D_0$ lifetimes for **B1** - **B10** and energy transfer efficiency (E) for **B2** - **B9**.

Compounds	544 nm ($^5D_4$) / μs	616 nm ($^5D_0$) / μs	E %
**B1**	1076.24	-	-
**B2**	1015.53	727.27	5.64
**B3**	952.58	646.27	11.49
**B4**	855.08	561.36	20.55
**B5**	834.85	560.45	22.43
**B6**	759.70	519.46	29.41
**B7**	750.27	530.60	30.29
**B8**	706.78	549.67	34.33
**B9**	644.97	496.32	40.07
**B10**	-	548.91	-

Supplementary Table 4. *R*-*G*-*B* chromaticity values of **B7** dispersions vary TMAO concentrations**.**

[C] / mM	*R*	*G*	*B*	*R/(G+B)*
0	201	165	148	0.642
0.20	218	183	153	0.649
0.40	228	196	152	0.655
0.60	238	197	160	0.667
0.80	244	200	162	0.674
1.0	238	192	157	0.682
1.19	238	181	161	0.696
1.38	225	166	153	0.705
1.57	232	169	157	0.712
1.76	215	138	156	0.731
1.96	210	156	125	0.747
3.85	220	140	118	0.853
5.66	216	109	119	0.947
7.41	247	111	125	1.047
9.09	250	106	121	1.101
10.7	254	102	114	1.176
12.3	248	91	117	1.192
13.8	248	92	115	1.198
15.3	237	84	114	1.197

Supplementary Table 5. ^{11}B nuclear magnetic parameters calculated at B3LYP/6-31G(d) level of H_2BIPA and $H_2BIPA\cdots TMAO$ which formed by bonding interactions of H_2BIPA and TMAO (model 1).

Model 1	Calculated C_Q (MHz)	Calculated σ_{iso} (ppm)
H_2BIPA	2.875	85.232
$H_2BIPA\cdots TMAO$	1.418	104.367

Where C_Q is the quadrupole coupling constant; σ_{iso} is isotropic chemical shielding factor. B atom is a tetra-coordination after bonding with TMAO and the spherical symmetry will increase, causing C_Q to decrease significantly, which does not agree with the observed small difference (4.4 MHz to 4.2 MHz), ruling out the possibilities of bonding interactions.

Supplementary Table 6. ^{11}B nuclear magnetic parameters of H_2BIPA and $H_2BIPA\cdots TMAO$ which formed by non-bonding interactions of H_2BIPA and TMAO obtained by structure optimization using Materials Studio (model 2).

Model 2	Calculated C_Q (MHz)	Calculated σ_{iso} (ppm)
**B1**	3.009	83.670
**B1**···TMAO	2.861	74.690

Where C_Q is the quadrupole coupling constant; σ_{iso} is isotropic chemical shielding factor. C_Q has little relationship with bonding but only with spatial position, and its variation trend is more reliable. C_Q of **B1** decreases a little with non-bonding interactions of TMAO, which agrees with experimental results (4.4 MHz to 4.2 MHz). The structure optimized by Materials Studio is at 0K, which is different from the structure at room temperature, causing the difference of experimental results and calculated results.

Supplementary Table 7. The lifetimes at 544 nm and 616 nm of **B7** aqueous dispersion with different concentration's TMAO excited at 254 nm.

[C] / mM	544 nm ($^5D_4$) / ms	616 nm ($^5D_0$) / ms	Lifetime ratio
0	0.431	0.189	0.439
1	0.399	0.191	0.479
2	0.375	0.197	0.525
3	0.354	0.203	0.573
4	0.337	0.209	0.620
5	0.327	0.210	0.642

Supplementary Table 8. EA and ICP-AES results for all compounds.

Compounds	Addition/mL	EA results (%)				ICP-AES results		Yield %
	Tb^{3+} : Eu^{3+} : Gd^{3+}	Elements	C	H	N	Tb	Eu	Based on H_2BIPA/H_2IPA
**B1**	1.00 : 0.00 : 0.00	Calcd	30.06	3.95	1.64	1	-	55
		Found	30.28	3.67	1.76			
**B2**	0.95 : 0.05 : 0.00	Calcd	30.11	3.95	1.65	0.87	0.13	52
		Found	30.31	3.54	1.56			
**B3**	0.90 : 0.10 : 0.00	Calcd	30.13	3.96	1.65	0.80	0.20	57
		Found	30.33	3.57	1.53			
**B4**	0.80 : 0.20 : 0.00	Calcd	30.15	3.96	1.65	0.76	0.24	48
		Found	30.24	3.82	1.50			
**B5**	0.75 : 0.25 : 0.00	Calcd	30.17	3.96	1.65	0.67	0.33	59
		Found	30.35	3.78	1.54			
**B6**	0.60 : 0.40 : 0.00	Calcd	30.25	3.97	1.65	0.44	0.56	51
		Found	30.41	3.82	1.51			
**B7**	0.50 : 0.50 : 0.00	Calcd	30.25	3.97	1.65	0.42	0.58	62
		Found	30.37	3.73	1.56			
**B8**	0.25 : 0.75 : 0.00	Calcd	30.28	3.98	1.65	0.35	0.65	53
		Found	30.41	3.72	1.60			
**B9**	0.10 : 0.90 : 0.00	Calcd	30.37	3.99	1.66	0.08	0.92	53
		Found	30.56	3.77	1.64			
**B10**	0.00 : 1.00 : 0.00	Calcd	30.39	3.99	1.66	-	1	58
		Found	30.47	4.12	1.82			
**B11**	0.00 : 0.00 : 1.00	Calcd	30.14	3.96	1.65	-	-	57
		Found	30.35	3.74	1.71			
**B12**	0.50 : 0.50: 0.00	Calcd	35.28	2.73	-	0.46	0.54	60
		Found	35.36	3.03	-			
**B12-Tb**	1.00 : 0.00 : 0.00	Calcd	35.02	2.74	-	1	-	63
		Found	35.29	2.98	-			

ICP-AES results show the normalized atomic ratio.

References

[S1] Committee, A. M. Recommendations for the definition, estimation and use of the detection limit. *Analyst* **112**, 199-204 (1987).

[S2] Ocque, A. J., Stubbs, J. R. & Nolin, T. D. Development and Validation of a Simple UHPLC-MS/MS Method for the Simultaneous Determination of Trimethylamine *N*-Oxide, Choline, and Betaine in Human Plasma and Urine. *J. Pharm. Biomed. Anal.* **109**, 128-135 (2015).

[S3] Mills, G. A., Walker, V. & Mughal, H. Quantitative Determination of Trimethylamine in Urine by Solid-Phase Microextraction and Gas Chromatography-Mass Spectrometry. *J. Chromatogr. B: Biomed. Sci. Appl.* **723**, 281-285 (1999).

[S4] Lakshmi, G. B. V. S.; Yadav, A. K.; Mehlawat, N. et al. Gut microbiota derived trimethylamine *N*-oxide (TMAO) detection through molecularly imprinted polymer based sensor. *Sci. Rep.* **11**, 1338 (2021).

[S5] Yu, H.; Geng, W.-C.; Zheng, Z. et al. Facile Fluorescence Monitoring of Gut Microbial Metabolite Trimethylamine N-oxide via Molecular Recognition of Guanidinium-Modified Calixarene. *Theranostics* **9**, 4624-4632 (2019).

[S6] Li Z. & Suslick, K. S. Ultrasonic Preparation of Porous Silica-Dye Microspheres: Sensors for Quantification of Urinary Trimethylamine *N*-Oxide. *ACS Appl. Mater. Interfaces* **10**, 15820-15828 (2018).

[S7] Zhang S.-Y.; Shi, W.; Cheng, P. et al. A Mixed-Crystal Lanthanide Zeolite-like Metal–Organic Framework as a Fluorescent Indicator for Lysophosphatidic Acid, a Cancer Biomarker. *J. Am. Chem. Soc.* **137**, 12203–12206 (2015).

[S8] Wang, Y.; Pei, H.; Jia, Y. et al. Synergistic Tailoring of Electrostatic and Hydrophobic Interactions for Rapid and Specific Recognition of Lysophosphatidic Acid, an Early-Stage Ovarian Cancer Biomarker. *J. Am. Chem. Soc.* **139**, 11616–11621 (2017).

[S9] Ikeda Masato; Yoshii T.; Matsui T. et al. Montmorillonite-Supramolecular Hydrogel Hybrid for Fluorocolorimetric Sensing of Polyamines. *J. Am. Chem. Soc.* **133**, 1670–1673 (2011).

[S10] Lim J. H.; Park J.; Oh E. H. et al. Nanovesicle-Based Bioelectronic Nose for the Diagnosis of Lung Cancer from Human Blood. *Adv. Healthcare Mater.* **3**, 360–3660 (2014).

[S11] Zhou, Y.; Hu, Q.; Yu, F. et al. A Metal–Organic Framework Based on a Nickel Bis(dithiolene) Connector: Synthesis, Crystal Structure, and Application as an Electrochemical Glucose Sensor. *J. Am. Chem. Soc.* **142**, 20313–20317 (2020).

[S12] Yin, H.-Q.; Wang, X.-Y. & Yin, X.-B. Rotation Restricted Emission and Antenna Effect in Single Metal–Organic Frameworks. *J. Am. Chem. Soc.* **141**, 15166–15173 (2019).

[S13] Sheydaei, O.; Khajehsharifi, H. & Rajab, H. R. Rapid and selective diagnose of Sarcosine in urine samples as prostate cancer biomarker by mesoporous imprinted polymeric nanobeads modified electrode. *Sensor Actuat B-Chem* **309**, 127559 (2020).
